# PKC-mediated phosphorylation governs the stability and function of CELF1 as a driver of EMT in breast epithelial cells

**DOI:** 10.1016/j.jbc.2024.107826

**Published:** 2024-09-27

**Authors:** Shebna Massey, Natee Kongchan, Yang Gao, Arindam Chaudhury, Emuejevoke Olokpa, Jason Karch, Anna Malovannaya, Chonghui Cheng, Xiang Zhang, Joel R. Neilson

**Affiliations:** 1 Department of Integrative Physiology, Baylor College of Medicine, Houston, Texas, USA; 2 Dan L. Duncan Comprehensive Cancer Center, Baylor College of Medicine, Houston, Texas, USA; 3 Lester and Sue Smith Breast Center, Baylor College of Medicine, Houston, Texas, USA; 4 Department of Molecular and Cellular Biology, Baylor College of Medicine, Houston, Texas, USA; 5 Verna and Marrs McLean Department of Biochemistry and Molecular Biology, Baylor College of Medicine, Houston, Texas, USA; 6 Department of Molecular and Human Genetics, Baylor College of Medicine, Houston, Texas, USA; 7 McNair Medical Institute, Baylor College of Medicine, Houston, Texas, USA

**Keywords:** epithelial–mesenchymal transition (EMT), metastasis, RNA binding protein, CELF1, protein phosphorylation, post-translational regulation, PKCα, PKCε

## Abstract

Epithelial to mesenchymal transition (EMT) is believed to be a principal factor contributing to cancer metastasis. The post-transcriptional and post-translational mechanisms underlying EMT are comparatively underexplored. We previously demonstrated that the CELF1 RNA binding protein is necessary and sufficient to drive the EMT of breast epithelial cells, and that the relative protein expression of CELF1 in this context was dictated at the post-translational level. Here, we elucidate the mechanism of this regulation. Mass spectrometric analysis of CELF1 isolated from mesenchymal MCF-10A cells identified multiple sites of serine and threonine phosphorylation on the protein, correlating with the increased stability of this protein in this cellular state. Analysis of phosphomimetic and serine/threonine-to-alanine phosphomutant variants of CELF1 revealed that these phosphorylation sites indeed dictate CELF1 stability, ubiquitination state, and function *in vitro*. Via co-immunoprecipitation and *in vitro* kinase assays, we identified the protein kinase C alpha and epsilon isozymes as the kinases responsible for CELF1 phosphorylation in a breast cell line. Genetic epistasis experiments confirmed that these PKCs function upstream of CELF1 in this EMT program, and CELF1 phosphorylation impacts tumor metastasis in a xenograft model. This work is the first to formally establish the mechanisms underlying post-translational control of CELF1 expression and function during EMT of breast epithelial cells. Given the broad dysregulation of CELF1 expression in human breast cancer, our results may ultimately provide knowledge that may be leveraged for novel therapeutic interventions in this context.

Epithelial to mesenchymal transition (EMT) is the loss of cell-cell adhesion and apical-basal polarity, along with the acquisition of fibroblast-like cell properties including spindle-shaped morphology and a gain in cell motility and invasiveness (1). During this transition, cells also exhibit phenotypic changes associated with differentiation, morphing from a differentiated phenotype to one that is more stem-like (2, 3). EMT is a normally occurring cellular process during embryogenesis, cell and tissue repair, and regeneration but is also a largely essential program underlying cancer metastasis (4, 5). More recent studies revealed EMT as a continuum of several different subtypes where cells are not only in epithelial or mesenchymal states but could exist in a “partial” E-M transitory phase, functioning like cancer stem cells (6). These cell trans-differentiation and stem-like characteristics in EMT enable the tumor to acquire intrinsic properties promoting chemoresistance, rendering conventional chemotherapies ineffective (7, 8). Due to commonalities between pathological and developmental EMT the regulatory mechanisms controlling this process are highly investigated. As a result, several key mediators of EMT, functioning at the levels of transcriptional and post-transcriptional regulation, have been characterized.

One of the most widely used model systems for the study of EMT is the *in vitro* response to treatment with the cytokine TGF-beta (TGF-β). Intracellular signaling originating from this stimulus leads to the increased activity of several transcription factors promoting downstream EMT programs, including δEF1 family proteins (ZEB1, ZEB2, and SIP1), TWIST, and SNAIL/SLUG (9, 10). However, in addition to the transcriptional response, broad changes in the post-transcriptional and translational regulatory landscape, mediated by RNA-binding proteins like hnRNPs, HuR, YB-1, RNA-binding motif proteins (RBMs), and TTP, as well as EMT-associated miRNAs like miR-10b, miR-577 and miR-200, have been documented (11, 12, 13, 14). Interestingly, while this litany of transcriptional and post-transcriptional players work together to mechanistically effect EMT, there is only partial redundancy within the immediate specific programs they individually drive (15). Thus, the need to identify additional regulators remains a salient one.

Using polyribosomal profiling of TGF-β mediated EMT in breast epithelial cells as an entry point, we previously identified the CELF1 (CUGBP and eELAV-like family member 1) RNA-binding protein as a key mediator of the translation of a select group of EMT driver mRNAs in the mesenchymal state (16). Within multiple *in vitro* experimental systems, CELF1 protein expression is both necessary and sufficient to drive EMT, and CELF1 loss-of-function in mesenchymal/de-differentiated breast cancer cell lines drives them back to a more epithelial/differentiated state. CELF1’s *in vitro* function is conserved in *in vivo* models, where it is necessary and sufficient to facilitate tumor progression in cell-derived xenografts, and analysis of primary breast cancer samples revealed increasing CELF1 protein expression solely as a function of tumor grade and lymph node involvement.

While CELF1 has been best characterized in the context of myotonic dystrophy type 1 (DM1) models (17, 18), a growing body of work has associated CELF1 overexpression with cancer proliferation, migration, invasion, and overall tumor aggressivity in multiple cancer models (19, 20, 21, 22). However, within the latter contexts, the mechanism by which CELF1 levels are themselves regulated is largely unexplored. In our own primary experimental model, we have shown that while both the relative levels of CELF1 mRNA and the association of this mRNA with polyribosomes are unchanged in a comparison of the epithelial and mesenchymal states, there is a marked increase of CELF1 protein in mesenchymal cells as compared to their epithelial counterparts. Treatment of breast epithelial cells with the proteasomal inhibitor MG132 results in a rapid increase in CELF1 protein expression in these cells, indicating that within the epithelial state mRNA encoding CELF1 is actively translated, but that the protein itself is immediately degraded (16).

The stability of CELF1 in our primary model is correlated with its phosphorylation state—while CELF1 derived from epithelial cells is largely unphosphorylated, CELF1 derived from mesenchymal cells is phosphorylated on both serine and threonine residues (16). Here, we describe the identification of the sites of phosphorylation on CELF1 in breast cells, characterize the impact of these sites on CELF1 stability and function, and *via* molecular genetics and biochemical approaches identify the kinases dictating this phosphorylation.

## Results

### CELF1 phosphorylation at serine and threonine sites is conserved in multiple EMT models

**Figure 1:**
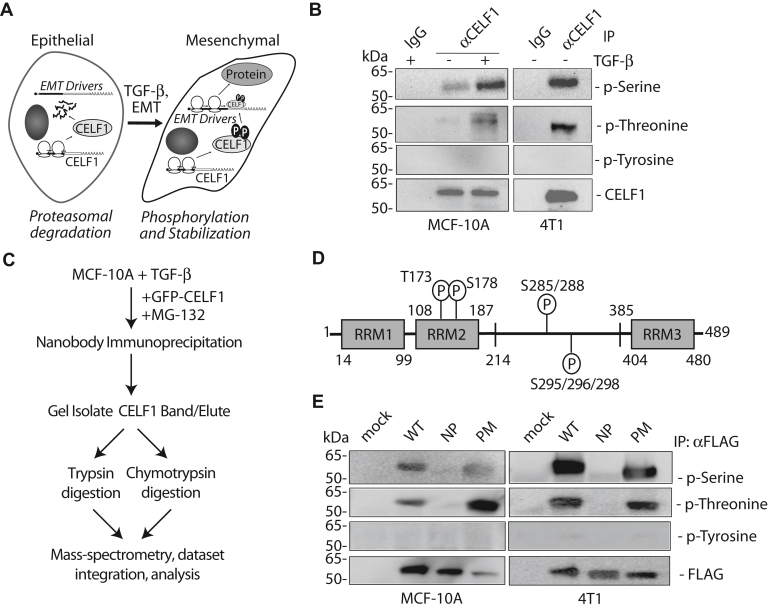
**CELF1 phosphorylation in breast and breast cancer cell lines.***A*, schematic of our previous findings (16). CELF1 is translated but immediately degraded in epithelial MCF-10A cells, becoming stabilized upon TGF-β treatment and transition to the mesenchymal state, where it promotes translation of EMT driver mRNAs. In this state, CELF1 is phosphorylated on serine and threonine residues. *B*, immunoblot demonstrating the phosphorylation state of endogenous CELF1 immunoprecipitated from the indicated cell lines. MCF-10A cells were treated with 5 ng/ml TGF-β for 72 h prior to collection of whole cell extracts and immunoprecipitation with either anti-CELF1 monoclonal antibody or control murine IgG as indicated. Blots were probed with the indicated antibodies. The CELF1 blot serves as the loading control. *C*, workflow schematic for mass-spectrometric identification of CELF1 phosphorylation sites. Parallel trypsin and chymotrypsin digestion and integration of the MS data were required to obtain robust coverage of the protein (Fig. S1). *D*, graphical depiction of serine/threonine phosphorylation sites on CELF1 identified *via* mass spectrometry. *E*, immunoblot of FLAG-tagged CELF1 expression constructs stably transduced into the indicated cell lines and induced *via* treatment with 0.1 μg/ml doxycycline for 72 h. During induction, MCF-10A cells were additionally treated with 5 ng/ml TGF-β for 72 h. Whole cell extracts were immunoprecipitated with anti-FLAG beads and the immunoprecipitate was probed with the indicated antibodies. Anti-FLAG (detecting CELF1) serves as the loading control. WT = wild type, NP = phosphomutant, PM = phosphomimetic. Figure 1, *B* and *E* representative of a minimum of three experimental replicates.

In our previous work, we established that CELF1 is unphosphorylated and immediately degraded in epithelial MCF-10A cells. However, when this cell line was treated with TGF-β and transitioned to a mesenchymal state, steady-state relative levels of CELF1 protein increased, promoting the translation of a cohort of EMT driver mRNAs. This increase in relative expression correlated with an increase in phosphorylation of CELF1 on serine and threonine residues (Fig. 1*A*, (16)). We first asked whether we might observe similar post-translational modification in the murine breast cancer cell line, 4T1 (23), where CELF1 protein is stably expressed and not subject to immediate degradation. CELF1 immunoprecipitated from this cell line was similarly phosphorylated on serine and threonine (but not tyrosine) residues, consistent with the notion that CELF1 stabilization by phosphorylation in breast cancer cell lines is evolutionarily conserved (Fig. 1*B*).

### Identification of CELF1 phosphorylation sites in breast epithelial cells

While phosphorylation sites on CELF1 have previously been described in a 293T cell model (24) we considered the possibility that the specific sites of phosphorylation on this protein might vary in cells derived from breast epithelium. We thus endeavored to identify the specific sites phosphorylated in our primary model system. To this end, we transiently transfected TGF-β treated MCF-10A cells with CELF1, coupled to EGFP *via* an N-terminal fusion. Using a camelid nanobody, we immunoprecipitated the fusion protein for mass spectrometric identification of sites that had undergone post-translational modification within the cells. A dearth of lysine and arginine residues in one region of CELF1 that we suspected to be phosphorylated (Fig. S1*A*) prompted us to digest our immunoprecipitates separately with both trypsin and chymotrypsin, collecting mass spectra for both digestions and re-integrating this data to obtain 97.7% overall coverage (Fig. 1*C*). Our analyses revealed phosphorylation at one threonine (T - T173) and six distinct serine (S - S178, S285, S288, S295, S296, S298) sites on CELF1 derived from mesenchymal MCF-10A cells (Figs. 1*D* and S1, *B* and *C*). As we suspected, while there was some overlap with the sites that had been previously described in 293Ts, five of these sites (S285, S288, S295, S296, S298) were unique to our model system. Informed by these data, we generated phosphomimetic (S→D, T→E) and phosphomutant (T173, S178, 283–285, 288, 292, 293, 295–302→A) variants of CELF1 for further analysis. We rationalized the mutation of additional S residues between 283 to 302 given the number of adjacent unmodified serine residues within this region and our observations in preliminary experiments that phosphorylation, presumably on these adjacent residues, could still be observed in mutant proteins in which the only the discrete serines that we had identified had been mutated to alanine (data not shown). Expression of the phosphomimetic and phosphomutant proteins in MCF-10A cells revealed that while the mutations abolished phosphorylation in the phosphomutant protein, signal indicating phospho-serine and phospho-threonine modification could still be observed in the phosphomimetic protein. These observations were consistent with the proteins expressed in the 4T1 line, again consistent with evolutionary conservation of the sites in cells derived from the breast epithelium (Fig. 1*E*). We suspected that the anti-phosphothreonine and anti-phosphoserine antibodies might recognize the phosphomimetic mutations, perhaps at a reduced affinity, but that this would be indistinguishable from native modifications in the context of immunoblot analysis. To test this notion, we expressed recombinant wild-type and phosphomimetic CELF1 in bacteria, purified these proteins, and analyzed them *via* immunoblot. As suspected, the phosphomimetic CELF1 derived from bacteria was robustly detected by these two antibodies (Fig. S1*D*), consistent with the notion that the immunoblot signal we observed for phospho-serine and phospho-threonine on protein derived from mammalian cellular extracts would be essentially indistinguishable from native modifications.

### CELF1 phosphomimetic mutations confer increased stability

**Figure 2:**
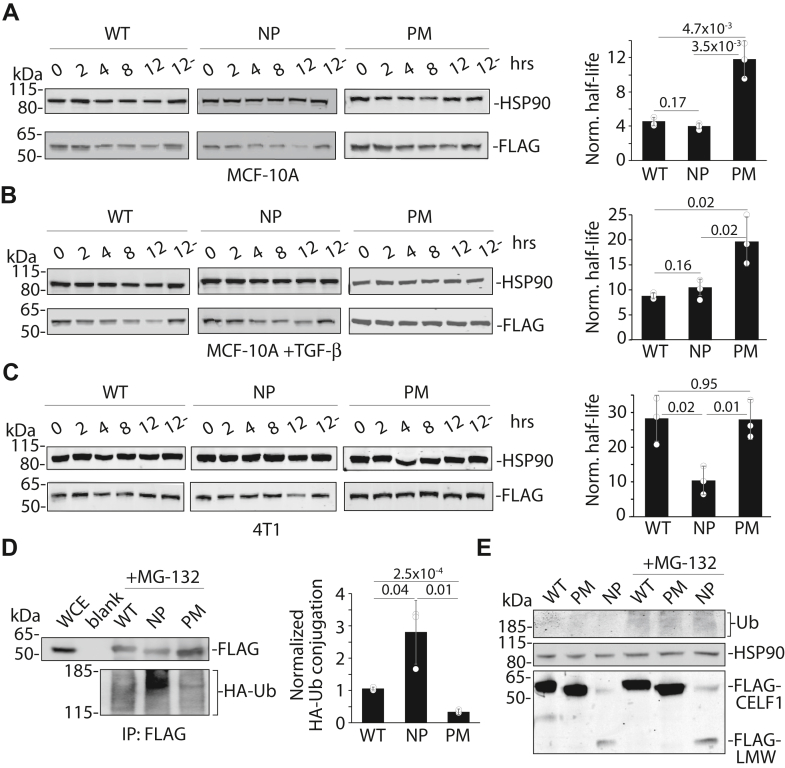
**CELF1’s phosphorylation sites confer altered stability and sensitivity to ubiquitination.** Immunoblots (anti-FLAG, *left*) and densitometric analysis (*right*) of the half-lives of the wild-type (WT), phosphomutant (NP), and phosphomimetic (NP) CELF1 expression constructs following treatment with 20 μg/ml cycloheximide (CHX) at the indicated timepoints (hours) in (*A*) untreated MCF-10A cells, (*B*) MCF-10A cells treated with 5 ng/ml TGF-β for 72 h and (*C*) 4T1 cells. *A-C*, For densitometric analysis, relative expression was calculated by normalizing FLAG signal to the HSP90 loading control, and then calculated as a function of normalized expression at the 0 h time point. The 12- indicates non-CHX treated control. Half-life was calculated as ln (2)/k. *D*, immunoblot (*left*) and densitometric analysis (*right*) of FLAG immunoprecipitates from whole cell extracts derived from HEK293T cells transiently co-transfected with expression constructs encoding HA-tagged ubiquitin and the indicated FLAG-tagged CELF1 variants. Twenty-four hours following transfection, the transfectants were treated with 20 μM MG132 for 8 h and harvested. Densitometric analysis of HA-Ub signal is normalized to each unmodified parent protein (*right*). Blank = buffer blank in lane. *E*, immunoblot of steady state expression of the indicated CELF1 rescue constructs in whole cell extracts of non-TGF-β-treated (epithelial) MCF-10A sublines incubated with 20 μM MG132 for 8 h. The ubiquitin (Ub) blot serves as a positive control for the potency of the MG132, and the HSP90 blot serves as a loading control. LMW = low molecular weight. The faint band migrating just under 50 kDa in the leftmost two lanes of the FLAG blot was not consistently observed. In all experiments, construct expression was induced *via* inclusion of 0.1 μg/ml doxycycline for 24 h prior to the indicated starting point. *p*-values from Student’s two-tailed *t* test indicated. Error bars indicate standard deviation. All immunoblots were quantified using ImageJ software, all figures are representative of a minimum of three experimental replicates.

Our previous work established a correlation between the phosphorylation state of CELF1 and the proteasomal degradation of this protein in our model system. To establish to what extent CELF1 phosphorylation might be causal to these changes in CELF1’s relative steady-state expression, we employed cycloheximide chase experiments in MCF-10A cells stably transduced with doxycycline-inducible lentiviral expression vectors (25) expressing FLAG-tagged wild-type and mutant proteins while simultaneously inducing the expression of an shRNA targeting the endogenous CELF1 mRNA transcript *via* the latter’s 3′ untranslated region (Fig. S2, *A* and *B*). Importantly, transcriptional induction of the three CELF1 constructs within their respective stably transduced sublines, as monitored by quantitative RT-PCR, was essentially equivalent (Fig. S2*C*). Consistent with phosphorylation conferring stability in the epithelial state, densitometric quantification of immunoblots for the affinity tag revealed that the half-life of the phosphomimetic mutant of CELF1 was roughly three times that of both the phosphomutant and the wild-type protein (Fig. 2*A*, 11.7 h *versus* 4.0 and 4.5 h, respectively) in untreated MCF-10A cells. The similarity in half-lives of the wild-type and phosphomutant proteins in the epithelial state is consistent with a lack of CELF1 phosphorylation within this state. While the half-lives of both wild-type and mutant proteins were increased in cells first treated with TGF-β for 72 h (the mesenchymal state), again the phosphomimetic mutant CELF1 was characterized by a roughly 2-fold greater half-life as compared to the phosphomutant and wild-type (Fig. 2*B*, 19.8 h *versus* 10.6 and 8.9 h, respectively). This comparative increase in half-lives in the mesenchymal state is consistent with a model in which CELF1’s rate of degradation is reduced within this state, perhaps reflecting decreased activity of the machinery dictating CELF1’s degradation within this context. Analysis of the three proteins in the murine 4T1 cell line again revealed a longer half-life for the phosphomimetic as compared to the phosphomutant (Fig. 2*C*, 27.8 h *versus* 10.3 h), although in this context the half-life of the wild-type CELF1 (28 h, again Fig. 2*C*) was essentially indistinguishable from the phosphomimetic. Nonetheless, this finding is consistent with the notion that the phosphorylation of CELF1 on the residues that we identified *via* mass spectrometry confers increased relative stability in both human and murine breast cell lines. Taken together, these data support a model in which basal CELF1 stability is increased in mesenchymal breast cells as compared to the same cells in an epithelial state, that CELF1 phosphorylation on specific residues confers increased relative stability in both of these contexts, and that the latter effect is evolutionarily conserved.

While these experiments were informative regarding CELF1 stability *per se*, they did not formally address the notion that CELF1 phosphorylation impacts the sensitivity of this protein to degradation by the proteasome. To directly establish this, we transiently transfected our stably transduced MCF-10A sublines with hemagglutinin-tagged ubiquitin (HA-Ub), unfortunately finding that the latter construct was lethal to our primary model system (data not shown). We thus examined basal ubiquitination of each of the three proteins upon transient transfection into HEK293T cells, treating transductants with the proteasome inhibitor MG132 for a period of 8 h prior to making whole cell extracts and immunoprecipitating each of the proteins *via* their FLAG affinity tag. We assessed HA-Ub signal within our immunoprecipitates *via* immunoblot and densitometric analysis. Consistent with our model, steady-state ubiquitination of the phosphomutant CELF1 was roughly five times that of the phosphomimetic, with the steady-state ubiquitination of the wild-type CELF1 protein falling in between these two mutants (Fig. 2*D*). These results indicate that in HEK293T cells, mutation of the specific serine and threonine residues that we observed to be phosphorylated in MCF-10A cells confers altered sensitivity to ubiquitin modification.

We have previously shown that treatment of epithelial MCF-10A cells with MG132 results in increased expression of endogenous CELF1 protein, after 8 h of treatment rising to the level of CELF1 expression in MCF-10A cells induced to undergo EMT with TGF-β treatment for 72 h (16). We thus asked whether this treatment would increase the steady-state levels of our CELF1 phosphomutant to match the levels of the wild-type and phospho-mutant CELF1. Unexpectedly, while treatment with MG132 for 8 h resulted in a consistent increase in phosphomutant expression within epithelial MCF-10A cells relative to untreated controls, this increase in this context was modest and did not result in expression comparable to the wild-type and phosphomimetic protein (Fig. 2*E*). Interestingly, a second lower-molecular weight species was consistently detected in extracts derived from the phosphomutant subline, with the molecular weight of this species suggesting a specific N-terminal degradation intermediate derived from proteolytic cleavage within CELF1’s intrinsically disordered region. Like the full-length phosphomutant CELF1, the visibility of this species modestly yet consistently increased following proteasomal inhibition with MG132. We concluded that mutation of these specific phosphorylation sites within the CELF1 protein renders the protein vulnerable to both proteolytic cleavage and proteasomal degradation, and that inhibition of the proteasome was not sufficient to promote expression of the phosphomutant CELF1 protein at levels comparable to wild-type or phosphomimetic protein within the MCF-10A model system.

### Mutation of CELF1 phosphorylation sites functionally impacts mesenchymal characteristics

**Figure 3:**
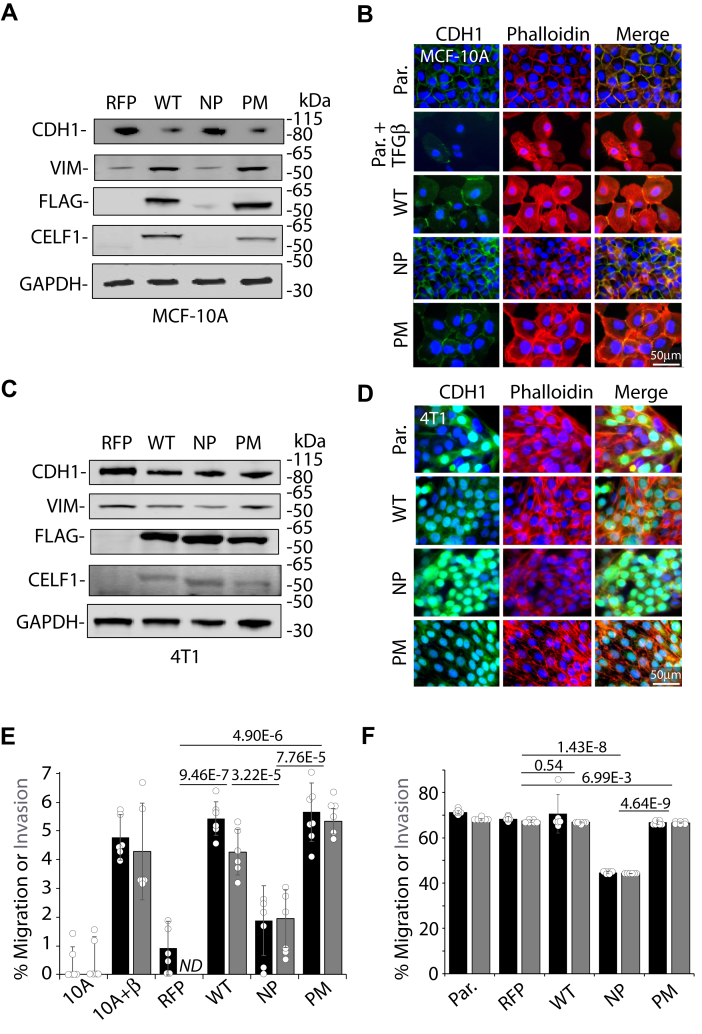
**CELF1’s phosphorylation sites impact CELF1’s efficacy in promoting the mesenchymal state.***A*, immunoblot of epithelial (CDH1) and mesenchymal (VIM) markers upon induction of wild-type (WT), phosphomutant (NP) and phosphomimetic (PM) CELF1 in stably transduced MCF-10A cells *via* induction with 0.1 μg/ml doxycycline for 72 h. *B*, immunofluorescence of E-cadherin (CDH1, *green*) and actin (Phalloidin, *red*) subcellular distribution upon induction of the indicated CELF1 variants, again using 0.1 μg/ml doxycycline for 72 h. DAPI nuclear counterstain is blue. E-cadherin and phalloidin distribution in untreated and TGF-β-treated (72 h) non-transduced parental cell lines are presented for reference. *C*, as (*A*), with induced expression of indicated variants in 4T1 cells. *D*, as (*B*), with induced expression of indicated variants in 4T1 cells. Again, the non-transduced parental cell (Par.) is presented for reference. *E*, transwell migration (*black bars*) and invasion (*grey bars*) in MCF-10A cells following induction of indicated CELF1 variants (0.1 μg/ml doxycycline) or for TGF- β treatment (5 ng/ml – positive control) for 72 h. RFP indicates cells stably transduced with a vector encoding inducible RFP and a control shRNA targeting β-galactosidase. Values normalized to untreated parental MCF-10A cells. *F*, as (*E*), with induction *via* 0.1 μg/ml doxycycline in 4T1 cells. Here the values represent the percent migration and invasion for each group. In (*E* and *F*), *p*-values from Student’s two-tailed *t* test indicated, the value shown represents the larger *p*-value of the two independent comparisons of migration and invasion. Error bars represent standard deviation. *ND* = not detected above baseline. All data representative of a minimum of three experimental replicates.

Having established that mutation of CELF1’s phosphorylation sites impacts the stability and steady-state levels of this protein, we turned to examine how these sites might in turn impact CELF1’s function within our model systems. We have previously demonstrated that overexpression of wild-type CELF1 in MCF-10A cells drives EMT as defined by changes in molecular markers, cell migratory capacity, and invasive capacity (16). We thus induced expression of each of the three CELF1 variants within their respective stably transduced sublines and assessed changes in the classical EMT markers E-cadherin (CDH1) and vimentin (VIM). As we had previously established, forced expression of wild-type CELF1 protein for a period of 72 h (here *via* doxycycline induction) led to a decrease in the relative expression of the epithelial marker E-cadherin concomitant with an increase in the relative expression of the mesenchymal marker vimentin as assessed by immunoblot (Fig. 3*A*). Ectopic expression of the phosphomimetic mutant of CELF1 resulted in similar changes. In contrast, the changes in E-cadherin and Vimentin expression observed in the wild-type and phosphomimetic sublines were not observed upon induction of the phosphomutant subline, with phosphomutant CELF1 protein again expressed at significantly lower levels as compared to the other two variants. We further assessed morphological changes resulting upon expression of the three proteins *via* immunofluorescence (26). In untreated MCF-10A cells or those in which the phosphomutant CELF1 was induced, E-cadherin and cytoskeletal actin (as visualized by phalloidin) were primarily localized to the cellular membrane and intracellular junctions (Figs. 3*B* and S3*A*), consistent with epithelial cellular morphology. In contrast, induction of either wild-type or phosphomimetic CELF1 in these cells resulted in a marked redistribution of E-cadherin and actin away from the membrane and intracellular junctions to the cytoplasm, this time consistent with a more mesenchymal cellular morphology and mirroring the changes occurring in MCF-10A cells treated with TGF-β.

In 4T1 sublines, modest decreases in the relative expression of vimentin could be qualitatively observed *via* immunoblot in cells expressing wild-type and phosphomutant CELF1 as compared to the subline expressing the phosphomimetic but were no clear differences in E-cadherin expression (Fig. 3*C*). However, immunofluorescent analysis of the 4T1 sublines expressing wild-type or phosphomimetic CELF1 revealed a cytoplasmic distribution of E-cadherin and actin somewhat analogous to that observed for TGF-β-treated MCF-10A cells (Figs. 3*D* and S3*B*). In contrast, 4T1 sublines expressing the CELF1 phosphomutant were characterized by a marked reorganization of E-cadherin to the perinuclear area ^27^. However, in this latter subline there was also a clear re-organization of actin to the cellular membrane, where co-localization with E-cadherin could be observed. The cells expressing the phosphomutant were markedly more rounded and less spindle-shaped than either control cells or cells expressing wild-type or phosphomimetic CELF1.

We next turned to examine whether the differences we observed in EMT marker expression and cell morphology upon CELF1 knockdown and wild-type or mutant re-expression would translate to analogous functional differences in transwell assays. As we had previously described (16), forced expression of wild-type CELF1 in untreated MCF-10A cells increased the relative migration and invasion capacity of these cells as compared to controls (Fig. 3*E*). While this increase was mirrored in the context of forced expression of the CELF1 phosphomimetic mutant, forced expression of the CELF1 phosphomutant only marginally increased cell migration as compared to controls, and this increase did not reach statistical significance (Fig. 3*E*). We observed complementary cellular behavior when examining the mesenchymal 4T1 cell line – while knockdown of CELF1 and rescue with either wild-type or phosphomimetic CELF1 resulted in effectively no change in cellular migration or invasion, rescue with the phosphomutant CELF1 resulted in significantly decreased migratory and invasive capacity (Fig. 3*F*). Importantly, knockdown of *CELF1* without rescue impeded both migration and invasion in both MCF-10A and 4T1 cell lines, and MTT assays revealed no significant differences in representation of viable cells among the individual sublines within these assays (Fig. S4). Taken together, our results are consistent with the conclusion that CELF1 phosphorylation on the discrete sites identified in MCF-10A cells is important for efficacy in either driving or maintaining a more mesenchymal cellular state in breast cells, and that the function of this phosphorylation within this context is again evolutionarily conserved.

### PKCα and PKCε phosphorylate CELF1 to drive EMT in MCF-10A cells

**Figure 4:**
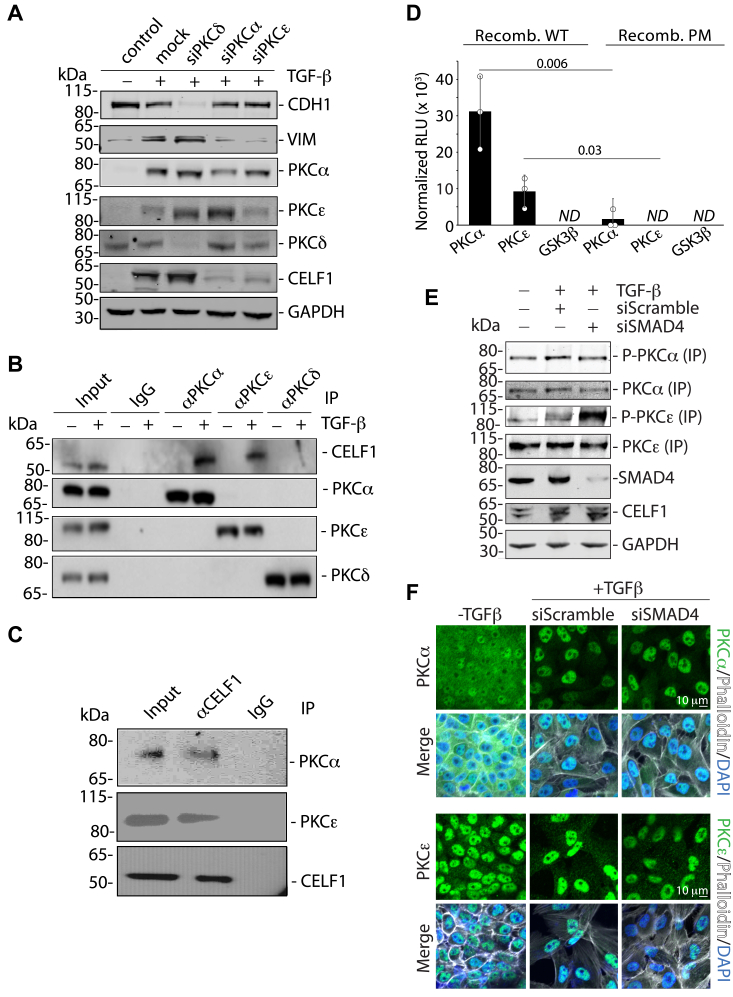
**PKCα and PKCε associate with CELF1 in cellular extracts and directly phosphorylate CELF1 *in vitro*.***A*, immunoblots showing relative expression of E-cadherin (CDH1), Vimentin (VIM), PKCα, PKC δ (specificity control), PKC ε, and CELF1 (all endogenous) following siRNA mediated knockdown of the indicated gene products and TGF-β treatment (5 ng/ml) of MCF-10A cells for 72 h. GAPDH is a loading control. *B*, immunoblot analysis of immunoprecipitations using the indicated antibodies from TGF-β-treated (as above) MCF-10A cytoplasmic extracts. *C*, immunoblot analysis of anti-CELF1 immunoprecipitations from TGF-β-treated (as above) MCF-10A cytoplasmic extracts. *D*, *in vitro* kinase assay using recombinant wild-type (WT) or phosphomimetic (PM) as substrate. GSK3β is included as a specificity control. Relative Light Units (RLU) normalized to standardized positive control from Promega (Cat. TM313). *p*-values from Student’s two-tailed *t* test indicated. Error bars denote standard deviation. *ND* = not detected above baseline. *E*, immunoblot analysis of an activating phosphorylation mark on PKCα and PKCε, both immunoprecipitated from untreated MCF-10A cells or cells transfected with 20 nM of the indicated siRNAs and then treated with TGF-β (5 ng/ml) for 72 h. Immunoblots of relative SMAD4, CELF1, and GAPDH (loading control) expression within the corresponding whole cell extracts is also shown. *F*, confocal immunofluorescence analysis (Nikon A1) of the subcellular localization of PKCα (*green, top*) and PKCε (*green, bottom*) in untreated MCF-10A cells or cells transfected with 20 nM of the indicated siRNAs and then treated with TGF-β (5 ng/ml) for 72 h. The cells are stained with Phalloidin (*white pseudocolor*) and DAPI (*blue*) for contextual visualization. All results are representative of a minimum of three experimental replicates. For experiments employing RNAi knockdown (*A*, *E* and *F*), the results are representative of a minimum number of three experimental replicates using each of two distinct siRNAs for each gene target.

Having established that the sites on CELF1 post-translationally modified by phosphorylation in MCF-10A cells played a role in CELF1 stability and function, we next turned to identify candidate kinases responsible for this phosphorylation. Synthesizing that Protein Kinase C alpha and Beta II (PKCα and PKCβII) have previously been implicated in CELF1 phosphorylation in models of Type 1 Myotonic Dystrophy (DM1) (17, 27), and that PKCα has long been known to impact MCF-10A cell morphology and motility (28), we reasoned that this or related kinases may play a similar role in the context of breast cells. We thus undertook a targeted screen, individually knocking down each member of the PKC family *via* siRNA in MCF-10A cells to determine how this manipulation might impact TGF-β-mediated EMT in our primary model system. As analyzed by immunoblot of CELF1 and EMT markers, knockdown of PKCα in TGF-β-treated MCF-10A cells essentially abolished both EMT and CELF1 expression (Fig. S5*A*), consistent with previously described observations in MCF-10A cells (28) and suggesting a relationship somewhat functionally analogous to that previously described in DM1 models (17, 27). Interestingly, knockdown of the PKC epsilon isoform (PKCε) had similar effects, again abolishing expression of CELF1 protein, and robustly repressing the shift in EMT marker expression that was observed in control cells. To validate our limited-scale screen, we repeated the knockdown of PKCα, PKCε, and PKC delta (PKCδ - as a specificity control) in TGF-β-treated MCF-10A cells, again observing disruption of EMT upon PKCα and PKCε (but not PKCδ) knockdown and confirming knockdown of each of the three kinases (Figs. 4*A* and S5*B*).

We next asked whether we could establish a physical interaction between CELF1 and each of these kinases. Immunoprecipitation of either PKCα or PKCε from cytoplasmic cellular extracts derived from TGF-β-treated MCF-10A cells revealed co-immunoprecipitation of CELF1 with these two kinases, an association that was not observed in untreated MCF-10A cells (Fig. 4*B*). CELF1 did not co-immunoprecipitate with PKCδ in either context. The association of CELF1 with PKCα and PKCε in TGF-β-treated MCF-10A cells was further validated by reverse co-immunoprecipitation (Fig. 4*C*). Importantly however, these experiments did not formally demonstrate that the interaction between CELF1 and these two kinases was either direct or productive.

To address this uncertainty, we performed *in vitro* kinase assays using purified recombinant proteins. Mirroring our functional observations in EMT assays, CELF1 was readily phosphorylated by PKCα, and phosphorylated by PKCε as well, albeit to a lesser extent (Fig. 4*D*). This level of phosphorylation was significantly diminished in *in vitro* kinase assays using purified recombinant phosphomimetic CELF1, consistent with the notion that the phosphorylation sites identified *via* mass spectrometry were indeed the sites being phosphorylated by these kinases in our *in vitro* assay. No phosphorylation above background was observed in the assays when purified Glycogen Synthase Kinase 3-beta (GSK3β) was used as a specificity control. Taken together, this set of experiments is consistent with a model that PKCα and PKCε directly phosphorylate CELF1 in MCF-10A cells, potentiating its function in promoting TGF-β mediated EMT.

We next explored the impact of TGF-β signaling on PKCα and PKCε activity, postulating that any changes in activity or localization arising from a canonical transcriptional response to TGF-β signaling would be impeded by disruption of *SMAD4* expression. Via immunoprecipitation, we first examined PKCα and PKCε activity in response to TGF-β treatment, probing these two proteins *via* a pan-phospho-PKC antibody (D6Y3D) specific for an activating phosphorylation mark (Figs. 4*E* and S6*A*). While no change in PKCα phosphorylation was observed following TGF-β treatment, we observed a modest increase in the activating phosphorylation mark upon PKCε in this context. Surprisingly, siRNA-mediated knockdown of SMAD4, while having no effect on the activating phosphorylation mark on PKCα, resulted in a relative increase of this mark upon PKCε - the opposite of what might have been expected. Also surprisingly, the relative increase in CELF1 expression occurring in response to TGF-β treatment was unaffected in the context of SMAD4 knockdown, strongly suggesting that this increase in relative expression is independent of a canonical TGF-β transcriptional response.

Given these unexpected results, we next employed confocal immunofluorescence to determine whether TGF-β treatment might impact the subcellular localization of PKCα and PKCε (Figs. 4*F* and S6*B*). PKCα was characterized by a very ubiquitous/diffuse staining pattern in untreated MCF-10A cells. However, upon TGF-β treatment, this staining robustly relocalized to the nucleus of the treated cells. In contrast, PKCε was present in both the nuclear and cytoplasmic compartments, with the majority of the signal localized the nucleus. This distribution remained consistent following TGF-β treatment. Again, somewhat surprisingly, SMAD4 knockdown had no impact on localization of either kinase. We concluded that TGF-β treatment results in an increase of PKCε activity and a reorganization of PKCα to the nucleus, and that these two events occur independently of a canonical TGF-β transcriptional response.

**Figure 5:**
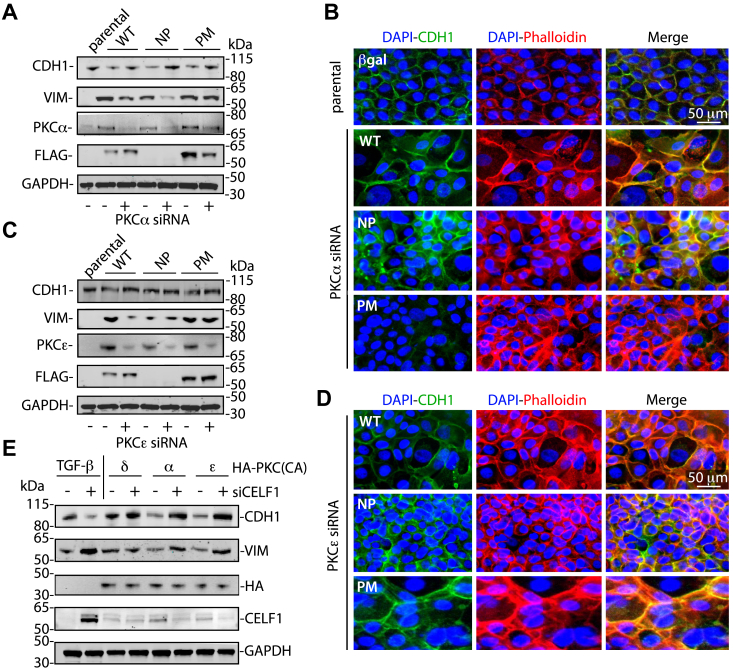
**PKC knockdown does not impact CELF1’s ability to drive EMT in MCF-10A cells.***A*, immunoblot of epithelial (CDH1) and mesenchymal (VIM) markers upon knockdown of PKCα with of siRNA, followed by induction of wild-type (WT), phosphomutant (NP) and phosphomimetic (PM) CELF1 with 0.1 μg/ml doxycycline for 72 h in stably transduced MCF-10A cells. *B*, Immunofluorescence of E-cadherin (CDH1, *green*) and actin (Phalloidin, *red*) subcellular distribution upon siRNA-mediated knockdown of PKCα followed by induction (as above) of the indicated CELF1 variants, DAPI nuclear counterstain is blue. *C*, as (*A*), knocking down PKCε rather than PKCα. *D*, as (*B*), again knocking down PKCε rather than PKCα. GAPDH serves as loading control for (*A* and *C*), while parental epithelial (non-TGF-β-treated) parental MCF-10A cells. *E*, immunoblot of epithelial and mesenchymal markers 72 h post-co-transfection of MCF-10A cells with the indicated HA-tagged catalytically active PKC mutants and 20 nM control siRNAs or siRNAs targeting endogenous CELF1. All figures representative of a minimum of three experimental repeats with each of two distinct siRNAs for each gene target.

We next turned to a molecular genetic approach, utilizing genetic epistasis assays in our primary model to determine to what extent PKCα, PKCε, and CELF1 were necessary and sufficient to drive EMT in our primary model system. As previously observed (Fig. 3*A*), forced expression of wild-type or phosphomimetic CELF1 effectively drove EMT in MCF-10A cells, whether assessed by immunoblot or immunofluorescent analysis of E-cadherin and actin subcellular localization, whereas forced expression of phosphomutant CELF1 did not drive these changes. The siRNA-mediated knockdown of neither PKCα (Figs. 5, *A* and *B*, S7, *A* and *B*, and S8) nor PKCε (Figs. 5, *C* and *D*, S7, *C* and *D*, and S8) significantly ameliorated wild-type or phosphomimetic CELF1’s ability to drive EMT in these contexts, consistent with CELF1 functioning genetically downstream of these kinases in the EMT process. Consistent with a model in which these PKC isozymes differentially regulate CELF1 during EMT, our data also revealed an increase in the relative expression of both PKCα and PKCε proteins occurring as a function of EMT in these experiments, with the level of increase correlating with observed changes in the canonical EMT markers upon forced expression of each of the CELF1 constructs. In the converse experiment, catalytically active mutants of PKCα, PKCε, and PKCδ (again, as a specificity control) were co-transfected into MCF-10A cells together with control siRNAs or siRNAs targeting endogenous CELF1. Curiously, transfection of MCF-10A cells with these catalytically active PKC isoforms resulted in significant toxicity, which could be largely ameliorated *via* inclusion of 5 μM of the lipid oxidation inhibitor Ferrostatin-1 in the transfection. In addition, while the catalytically active PKC isoforms were robustly expressed at 24 h post-transfection, the expression of these isoforms was significantly curtailed by 72 h, perhaps analogously mirroring the phenomenon by which sustained activation by phorbol esters leads to dowregulation of PKCs and termination of signaling. Even so, modest decreases in both E-cadherin and vimentin were observed upon transfection of catalytically active PKCα or PKCε, and these were ameliorated upon CELF1 knockdown (Figs. 5*E* and S7*E*). These results support our core model, in which PKCα and PKCε are the kinases that directly phosphorylate CELF1 on the residues modified in mesenchymal MCF-10A cells, and that CELF1 functions genetically downstream of these two kinases in this context.

We next returned our attention to the issue of the CELF1 phosphorylation impacting its relative stability. Within our model, there are two distinct possibilities: in the first, CELF1 phosphorylation during EMT could be an active dynamic process, in which CELF1 is inherently vulnerable to active phosphatases within the mesenchymal state and requires continued PKCα- and PKCε-mediated phosphorylation to maintain its stability and relative expression. In the second possibility, phosphorylation of CELF1 by PKCα and PKCε is more of a licensing event, in which phosphorylated CELF1 is not inherently vulnerable to phosphatases and degradation. To differentiate between these two possibilities, we treated wild-type MCF-10A cells with 5 ng/ml TGF-β for 72 h to induce EMT and CELF1 expression, then blocking ribosomal translation with 40 μg/ml cycloheximide in the presence or absence of 0.5 μM of the PKC inhibitor sotrastaurin. Via immunoblot, we monitored relative CELF1 levels over the course of 8 h post-treatment, finding no significant difference in CELF1 stability between sotrastaurin-treated and untreated cells within this period (Fig. S9). These results are consistent with the latter of the two aforementioned possibilities, in which continued PKCα- and PKCε-mediated phosphorylation of CELF1 is not required to maintain CELF1’s stability in the mesenchymal state.

### CELF1 phosphorylation sites govern tumor metastasis *in vivo*

**Figure 6:**
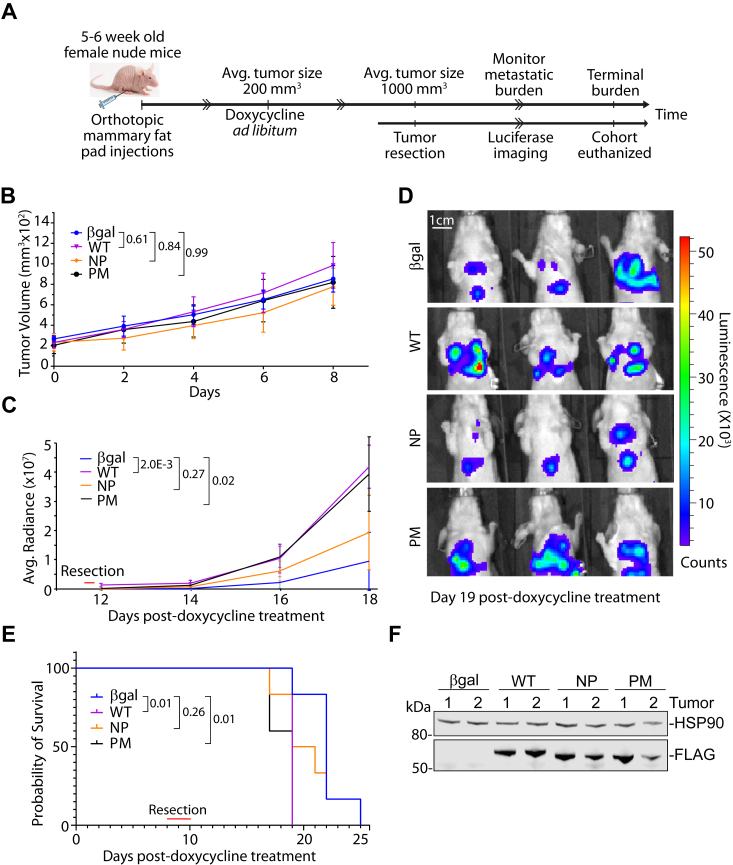
**CELF1 stability increases metastatic potential of primary tumors.***A*, schematic of experimental strategy. *B*, kinetics of growth of primary tumors initiated with the indicated inducible 4T1 sublines, following induction of *CELF1* knockdown/rescue with the indicated *CELF1* variant *via* 200 μg/ml doxycycline in drinking water provided *ad libitum* (n = 6 per group). Adjusted *p*-values from a Two-way ANOVA followed by Tukey’s test for multiple comparisons are indicated, error bars depict standard deviation. *C*, average radiance of lung metastases at indicated days post-induction. Indicated *p*-values derived from Student’s two-tailed *t* test of radiance on Day 18 for each indicated comparison, error bars depict standard deviation. *D*, Representative bioluminescence images in mice following primary tumor resection, 19 days post-induction. *E*, Kaplan-Meier plot of survival for each cohort following induction. The endpoint was humane euthanasia under the standard criteria of body score and labored breathing. *p*-values derived from Mantel-Cox Log-rank test for each indicated comparison. *F*, relative expression of CELF1 variant expression in representative primary tumors.

Having established the importance of CELF1’s phosphorylation sites on the stability and function of this protein *in vitro*, we turned to ask whether this function was conserved in an *in vivo* setting, following the experimental strategy outlined in (Fig. 6*A*). Briefly, we orthotopically injected the stably transduced 4T1 sublines into female nude mice, alongside an additional control subline expressing an RFP coding sequence with a control siRNA targeting Beta-galactosidase (β-gal). Once the primary tumors reached a size of ∼200 mm^3^, endogenous CELF1 and rescue with wild-type, phosphomimetic, or phosphomutant CELF1 was induced by introduction of doxycycline into the cohorts’ drinking water. In contrast to our previously published results utilizing constitutive knockdown without rescue or overexpression in human cell-derived xenograft models (16), no measurable differences were observed in the kinetics of primary tumor growth following induction of vector expression (Fig. 6*B*). The primary tumors were excised once they reached a size of ∼1000 mm^3^, at which point relative metastatic burden was actively monitored by vital bioluminescence imaging. In this secondary analysis, both wild-type and phosphomimetic CELF1 drove quantifiably higher metastatic burden as compared to the phosphomutant CELF1 and control line (Fig. 6, *C* and *D*). This metastatic burden was reflected in the overall fitness of the relative cohorts, with both wild-type and phosphomimetic CELF1 driving a significantly accelerated onset of morbidity as compared to the control and phosphomutant groups (Fig. 6*E*). Interestingly, within these experiments we observed relative expression of the phosphomutant CELF1 at levels essentially indistinguishable from levels of expression of the phosphomimetic and wild-type proteins (Fig. 6*F*). This raises the intriguing possibility that phosphorylation confers additional functionality to CELF1 beyond impacting its stability as observed in our *in vitro* experiments. In any case, these results are consistent with a model in which CELF1 phosphorylation state plays a key role on metastatic progression *in vivo*.

## Discussion

We had previously demonstrated that CELF1 is translated but immediately degraded by the proteasome in epithelial MCF-10A cells, but upon treatment of these cells with TGF-β and transition to a more mesenchymal state, CELF1’s relative stability increased concurrently with an increase in phosphorylation of this protein (16). However, the mechanism of this post-translational modification and whether it was causal to changes in stability remained to be explored.

Here we established that CELF1 phosphorylation is an event conserved in two distinct breast cancer cell lines and used our primary model system to identify the sites phosphorylated on CELF1 within this context. Using phosphomimetic and phosphomutant variants, of CELF1, we directly demonstrated that the phosphorylation sites that we identified on CELF1 impact the stability of this protein in multiple cell states and cell lines, also demonstrating that these sites impact sensitivity to ubiquitination and degradation. Consistent with these effects on stability and steady-state expression, we showed that the phosphomutant CELF1 is inefficient in driving or maintaining a more mesenchymal state *in vitro*, as well as metastatic progression *in vivo*, when compared to wild-type or phosphomimetic CELF1. We identified PKCα and PKCε as kinases playing a role in the EMT of the MCF-10A line, next showing that these kinases associate with CELF1 in the mesenchymal state and demonstrating direct phosphorylation of CELF1 in *in vitro* kinase assays with purified recombinant protein. We demonstrated that TGF-β signaling results in an increase in PKCε activity and a relocalization of PKCα to the nucleus, and that both of these events are independent of a canonical TGF-β transcriptional response. Finally, we tested the PKCα/ε and CELF1 functional relationship *via* a molecular genetic approach, confirming that CELF1 functions as an EMT driver downstream of PKC activity.

### CELF1 phosphorylation and stability

The impact of the CELF1 phosphorylation state on its stability/degradation has been shown in both DM1 models and models of liver cancer (17, 27, 29). Although phosphorylation sites conferring stability on CELF1 have been previously mapped and validated in the DM1 models (24), we speculated that there was some possibility that these sites may vary as a function of cell type or state. Indeed, by performing mass spectrometry on CELF1, expressed in the context of our primary model system, we were able to identify novel sites of phosphorylation. In comparison to these previous findings, only two common sites (T173 and S178) were identified. While Verma *et al.* did identify two sites in the C-terminus of the SP-repeats within CELF1’s intrinsically disordered region (S300, S302), our own data shows that within TGF-β-treated MCF-10A these repeats are more heavily phosphorylated, and that this occurs on serines (S178, S285, S288, S295–6, S298) distinct from those previously reported. These differences underscore the potential importance of *de novo* identification and/or confirmation of post-translational modifications in new cell types or states.

By employing phosphomimetic and phosphomutant variants of CELF1, we were able to directly establish that these phosphorylation sites confer altered stability to the CELF1 protein, irrespective of cell state or identity. Interestingly, in MCF-10A cells, while the half-life of the CELF1 phosphomimetic was consistently increased, the half-life of the CELF1 phosphomutant was essentially indistinguishable from wild-type CELF1, whether in more epithelial or more mesenchymal states. We speculate that this is a function of the permissivity of the regulatory environment within the two cellular states. Specifically, while the otherwise mutated sites are available within the wild-type protein in the more epithelial state, PKCα and PKCε do not associate with CELF1 in this context, thus the sites would remain unmodified, and the wild-type and phosphomutant CELF1 might thus be expected to be degraded at similar rates. Within the more mesenchymal state, one must also invoke the degradation machinery to make this argument. In this latter case, complete and constitutive phosphorylation is effectively forced in the phosphomimetic CELF1 by virtue of its substitutions, perhaps promoting additional stability as compared to the wild-type protein, where modification of these sites is likely to function in an equilibrium. The similar stabilities of the CELF1 phosphomutant and wild-type CELF1 here could then be a result of decreased activity of the machinery promoting CELF1 degradation in more mesenchymal cells. To our knowledge, the specific players directing the degradation of CELF1 in this context have not been identified, and we hope to identify these factors in future work.

Interestingly, and in stark contrast to our previous work demonstrating levels of CELF1 in epithelial MCF-10A cells comparable to mesenchymal MCF-10A cells upon proteasomal inhibition (16), here MG132 treatment did not elevate expression of the phosphomutant CELF1 to levels comparable to the expression of the wild-type and phosphomimetic proteins. At the same time, a distinct N-terminal proteolytic cleavage product specific to the phosphomutant was observed in this context (Fig. 2*E*). We favor the explanation that although the SP repeat region in which the bulk of modifications we identified *via* mass spectrometry is found within an intrinsically disordered region (IDR) of the CELF1 protein, the necessity of heavy serine-to-alanine substitutions to eliminate “next-neighbor” phosphorylation has somehow impeded translation of the phosphomutant protein, rendered it additionally vulnerable to degradation *via* a relevant or novel proteolytic event within CELF1’s IDR, or both. This reduces to some extent confidence that our functional experiments utilizing the phosphomutant CELF1 truly recapitulate what would be observed for a CELF1 in which phosphorylation was otherwise impeded. However, that full-length CELF1 phosphomutant could be readily detected in our model system, and that both this protein and its N-terminal cleavage product were increased upon MG132 treatment argues, against an immediate and complete proteolytic cleavage event. We thus conclude that the data assessing the relative stability of this mutant is a reasonable proxy, and in any case contend that the increase in stability of the phosphomimetic CELF1 relative to the wild-type protein fully supports the model that we propose. Additionally, our xenograft experiments clearly demonstrate that the phosphomutant CELF1 cannot drive metastasis to the extent driven by the wild-type and phosphomimetic proteins, even within the context of essentially equivalent relative expression. As previously mentioned, this raises the distinct possibility that CELF1 phosphorylation confers novel functions to the protein. We are excited to directly address this possibility in future work.

### CELF1 phosphorylation and function

The observed differences in relative stability among the three CELF1 variants translated directly to their efficacy in driving or maintaining a more mesenchymal state, underscoring the role that CELF1 phosphorylation and stability play in CELF1’s function in this context. In untreated MCF-10A cells, E-cadherin is robustly expressed and localized to cell-cell junctions. Similar to the changes that we have described for TGF-β treatment (16), overexpression of either wild-type or phosphomimetic CELF1 results in decreased expression of E-cadherin, as well as redistribution away from the membrane and these cell-cell junctions. The cells become less well-organized and there is an increase in the relative expression of the mesenchymal marker vimentin. While the phosphomimetic mutant of CELF1 effectively drives these changes, the phosphomutant is significantly less potent in this regard. These relative impacts on marker expression and cell morphology are conserved to functional assays, where while wild-type and phosphomimetic CELF1 effectively drive cellular migratory and invasive behavior in transwell assays, the changes in this behavior upon forced expression of the phosphomutant is again much less pronounced.

We employed MCF-10A cells as our primary model due to the facility of the model and the clear and robustly reproducible changes that can be observed upon manipulation, so it was relatively unsurprising that the differences we observed within the 4T1 model were to some degree more nuanced. In marker analysis within these cells, less robust qualitative changes could be observed in E-cadherin and vimentin expression upon knockdown of CELF1 and rescue with each of our three variants—indeed, the difference of expression of these two markers in cells in which knockdown had been rescued with phosphomutant CELF1 as compared to the other two variants was visible but quite modest. However, visualization of cell morphology, as well as the subcellular localizations of E-cadherin and actin in the context of these manipulations revealed a clearer picture, with E-cadherin and actin clearly showing a degree of re-organization to the cellular membrane. Accompanying this, and perhaps the clearest observation from these experiments, is a near-uniform nuclear/perinuclear localization of E-cadherin. This is consistent with previous descriptions of an active endocytic mechanism keeping E-cadherin from the membrane and cell-cell junctions (26, 30, 31, 32).

Our results in the 4T1 model perhaps better reflect the realities of EMT in an *in vivo* or disease setting, where it is difficult to classify cancer cells as purely epithelial or purely mesenchymal (33, 34). Indeed, where the cells reside on the spectrum between these two extremes comprises the histologically defined differentiation state (or grade) of a tumor, which remains an accurate key predictor of overall disease outcome (35), and intermediatory cell populations have been shown to be more tumorigenic and metastatic than those closer to either edge of this spectrum (33, 36). This kind of heterogenic cell population has also been shown to contribute towards chemoresistance due to due its stemness properties (37, 38). Interestingly, and in contrast to our previous results in a human cell-derived xenograft model (16), we found that induction of CELF1 knockdown and rescue with any of our three variant proteins had no impact on the kinetics of growth of a primary tumor derived from 4T1 cell implantation. This difference could be due to several reasons. Three of the most salient possibilities are: (i) that the former experiments were conducted in the context of constitutive CELF1 knockdown *a priori*, (ii) the inherent aggressivity of the 4T1 obscures robust effects in the timeframe between doxycycline-mediated induction and primary tumor resection, or simply, (iii) that there are as-yet-uncharacterized differences in how CELF1 manipulation influences the behavior of the two models. Nonetheless, even in this context both the wild-type and phosphomimetic CELF1 were clearly able to drive accelerated metastatic progression as compared to the phosphomutant, even though the latter protein was expressed at comparable levels within the experiment. Given that these were knockdown/rescue experiments, it is somewhat surprising that the kinetics of metastatic progression and onset of morbidity in the cohort in which the 4T1 cells expressed the CELF1 phosphomutant were indistinguishable from a cohort in which the cells expressed a control vector. Importantly the CELF1 phosphomutant was expressed at levels comparable to those observed for wild-type and phosphomimetic proteins in this context. We speculate that overexpression of the phosphomutant confers some level of functionality in the highly permissive 4T1 cellular environment, or perhaps that expression of this mutant did not push the cells far enough to the epithelial border of the differentiation spectrum to inhibit metastatic colonization and/or growth. These possibilities are clearly neither mutually exclusive nor exhaustive.

### PKCα and PKCε phosphorylate CELF1 in MCF-10A cells

Previous work on CELF1 phosphorylation in DM models (17, 19) steered us to examine the PKC family within our own context. Similar to these previous results, we found evidence that more than one PKC family member seems to be important for CELF1 phosphorylation in our model system—while the previous work demonstrated that PKCα and βII phosphorylate CELF1 in muscle cell lines (17), in the context of cells derived from the breast epithelium PKCα and PKCε appear to fulfill this role. While one might intuitively expect that the action of two related kinases in this context would be functionally redundant, RNAi knockdown of either of the two kinases individually impedes EMT of MCF-10A cells, indicating that they are both necessary for this process and consistent with a model of stepwise phosphorylation by each enzyme. PKCα belongs to the “classical” (Ca^2+^-dependent) and PKCε to the “novel” (Ca^2+^-independent) subclasses of the PKC family, meaning that their activity requires different cofactors and stimulatory signals (39). While we did demonstrate direct phosphorylation of purified CELF1 by each of these purified kinases *in vitro*, it is clearly possible that they each phosphorylate distinct sites on CELF1 necessary for stabilization and/or function. This is again consistent with a sequential mode of action on CELF1. Of course, these kinases likely have additional targets within the EMT program, and that the effect on EMT by RNAi knockdown of either of these kinases is most likely a function of the aggregate of these targets. Indeed, both PKCα and PKCε have long been implicated in breast cancer progression and the EMT of breast cancer cell lines (40, 41, 42, 43, 44), and an excellent body of work from the Rotenberg laboratory has firmly established α6-tubulin and CEP4 as direct targets of PKCα underlying robust changes in morphology and motility of MCF-10A cells upon stable overexpression of this kinase within them (28, 45, 46, 47, 48). We were surprised then when we observed that transient overexpression of constitutively active PKCα and PKCε within the same cell line resulted in significant toxicity, poor maintenance of ectopic expression, and only modest levels of EMT as monitored by marker expression (Fig. 5*E*) within the 72-h timeframe defining our standard experimental conditions. A key difference between our work and the work of Rotenberg’s group is that the latter has consistently employed stable transfectants in which wild-type rather than constitutively active PKCα is ovexpressed. It is of enormous interest to employ a variety of additional strategies in the future to dissect the contribution of PKCα, PKCε, PKCα′s previously established cytoskeletal targets, and CELF1 to EMT in this context, both defining the interplay among these players and evaluating potential connections between them and the MEK/ERK and FRA1 pathways (41, 43, 44). Drawing again from the DM1 literature, evidence does exist for PKC-independent pathways by which CELF1 may be regulated (17), providing fertile ground for additional discovery.

Treatment of MCF-10A cells with TGF-β results in an apparent increase in PKCε activity, concomitant with marked relocalization of PKCα to the nucleus. We were surprised by the finding that both of these events, and CELF1 stabilization itself, were unaffected by SMAD4 knockdown. This strongly suggests that these events are not secondary to a canonical TGF-β transcriptional response and that they occur in parallel to SMAD-dependent transcription *via* a parallel non-canonical TGF-β signaling. The nature of this non-canonical signaling is an exciting area for further exploration. In any case, our data indicate it likely that the nucleus is the site of CELF1 phosphorylation by PKCα. On one hand, this is intuitive given CELF1’s established role as a mediator of alternative splicing ^18,19^. On the other hand, for this phosphorylation to occur CELF1 must first transit to the nucleus from the ribosome. The protein’s active translation but immediate degradation in the epithelial state (16) poses a significant barrier to this transit. An initial phosphorylation by PKCε could in theory facilitate this initial event, given this kinase’s apparent activation upon TGF-β treatment and presence within the cytoplasm. However, the predominant localization of PKCε to the nucleus would also support a nuclear site of action for the kinase. In this latter case, one must invoke additional actors mediating CELF1’s initial transit to the nucleus Additional work is required to more concretely define this first step.

**Figure 7:**
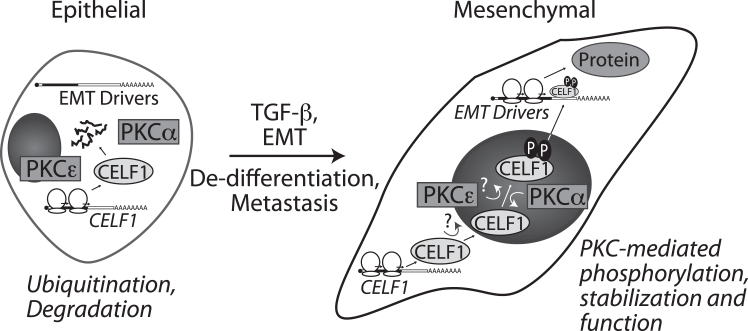
**Working model for CELF1 regulation *via* phosphorylation by PKCα and ε in breast epithelial EMT and metastasis.** Our data support a model in which TGF-β stimulation of breast epithelial cells results in a SMAD4-independent re-localization of PKCα to the nucleus concomitant with phosphorylation and stabilization of CELF1. Given the strong nuclear localization of PKCα in TGF-β-treated cells, it is likely that PKCα phosphorylates CELF1 occurs in the nucleus. PKCε′s site of action is less clear given that it is localized both to the nucleus and cytoplasm in both the epithelial and mesenchymal states. Ultimately though, our data strongly support the notion that phosphorylation of CELF1 protein by PKCα and PKCε is a licensing event that confers stability upon CELF1 and engenders the ability of this protein to facilitate the translation of EMT driver RNAs, thereby promoting and maintaining the mesenchymal/de-differentiated state.

From a clinical perspective, the differentiation state of the tumor is a product of the mutations engendering it. Once identified, this tumor is resected, precluding additional de-differentiation arising from additionally acquired mutations or other factors. However, even with well-differentiated tumors, circulating tumor cells may be observed in the bloodstream of patients (49), indicating that potential metastases have been seeded and may pose a future risk. From a therapeutic standpoint then, it is desirable to drive these latent metastases back to a more epithelial state, which may re-confer sensitivity to first- and second-line therapeutics (33, 50, 51). From our findings, we propose a model in which under TGF-β induction, CELF1 functions downstream of PKCα and PKCε to drive EMT and metastasis in breast cancer cells (Fig. 7). Our data raise the possibility that therapeutic intervention simultaneously targeting both PKCα and PKCε might have some therapeutic benefit to patients, although it will be an important next step to formally demonstrate that the function of these specific isoforms is broadly conserved in additional models of breast cancer. It is also worth noting that several therapeutic interventions targeting the PKC family have been assessed in the context of breast, colorectal, and lung cancers (52, 53). However, given the homology within the PKC family, the corresponding lack of specificity for small-molecule inhibitors of the family (44, 54), and the pleiotropic function of the family *in vivo*, a therapeutic selectively targeting subsets of these kinases would need to be developed, which remains a daunting task (55, 56). Interestingly, CELF1 misexpression has been observed in several types of solid tumors, (16, 19, 57, 58, 59, 60). While the discordance between the PKC isoforms at play in DM1 and our own model suggest that a similar discordance may be associated with other tissues, perhaps CELF1 misexpression has the potential to be a common target that might ultimately be exploited.

## Experimental procedures

### Cell culture and treatments

All cell lines were cultured and maintained under standard conditions (humidity-controlled incubator with 5% CO_2_ at 37° C), and routinely tested for *mycoplasma* contamination by the MD Anderson Cytogenetics and Cell Authentication Core. The human mammary breast epithelial cell line MCF-10A and human embryonic kidney cell line HEK293T were obtained from ATCC. MCF-10A cells were cultured in DMEM/F-12 (Dulbecco's Modified Eagle Medium/Nutrient Mixture F-12) from Gibco (Cat. 11320033), supplemented with 5% heat-inactivated horse serum (HS) from ThermoFisher (Cat. 16050122), 20 ng/ml epidermal growth factor (EGF - ThermoFisher, Cat. PHG0313), 0.5 mg/ml hydrocortisone (Sigma H-0888), 100 ng/ml cholera toxin (Sigma, Cat. C-8052), 10 μg/ml insulin (Sigma, Cat. I-1882), and 1% penicillin-streptomycin (Gibco Cat. 15140122). Both the 293T cell line and the murine breast carcinoma cell line 4T1 were cultured in Gibco high glucose DMEM (Cat. 11965084) with 10% heat-inactivated FBS and 1% Pen/Strep. MCF-10A cells stably transduced with conditional lentiviral vectors were maintained in the same basal media recipe as for MCF-10A cells, except HS was replaced with FBS to prevent HS-tetracycline-induced expression of the conditional promoter.

### Knockdown and over-expression experiments

Silencer Select siRNAs from ThermoFisher Scientific were used for knockdown of: PKCα (s11094, 11,092), PKCε (s11101, s11102), GSK3β (s6240, s6241), SMAD4 (s502547, s502548), and CELF1 (ID: s20954, s20954). CELF1 knockdown was additionally achieved *via* transfection of a previously described and specificity-validated laboratory plasmid driving expression of a shRNA targeting this message (16). The constitutively active PKC-alpha (#21234), -epsilon (#21242), and -delta (#16388) were purchased from Addgene. Briefly, 20 nM of siRNA was transfected using Lipofectamine LTX (Invitrogen, Cat. A12621). Cells were harvested 48 to 72 h post-transfection as indicated. Respective knockdowns were confirmed in immunoblotting experiments by probing for the knockdown gene compared to the controls. Ferrostatin-1 (Millipore Sigma SML0583) was included at 5 mM to extend cell viability in cells transfected with constitutively active PKC constructs.

### Plasmid construction, transfections, and transductions of lentiviral expression system

We generated the CELF1-mutants *via* site-directed mutagenesis using the Quikchange XL site-directed mutagenesis kit from Agilent (Cat. 200523). For the CELF1-PM, all serine (S) sites were mutated to aspartate (D), and all threonine (T) sites to glutamate (E). And, for CELF1-NP, all S/T sites were mutated to alanine (A), along with additional deletion of the SP-repeat linker region (Fig. S1 *A*). We modified the *pInducer-*10 lentiviral vector (25) for CELF1 knockdown and knockdown/rescue. Restriction digested *pInducer-10* backbone with *AgeI* and *NotI* was recombined with the CELF1 (WT, NP, PM) inserts with NEBuilder HiFi DNA Assembly Master Mix (NEB Cat. E2621S) and transformed into NEB-5-alpha competent cells (Cat. C2987H). Briefly, respective CELF1-mutant *pInducer-10* lentivirus was packaged in 293T cells. Cell-free supernatant was collected at 48 and 72 h post-transfection and applied to MCF-10A and 4T1 cell lines at ∼80 to 90% confluency. Transduced cells were selected with puromycin for >10 days at 2 μg/ml for MCF-10A, and 1 μg/ml for 4T1 cells. Expression of an N-terminal FLAG-tag on each coding sequence was confirmed upon doxycycline induction *via* immunoblot. For HA-Ub experiments, 293T cells were transiently co-transfected with *pInducer-10* WT, NP and PM CELF-variants, individually. After 48 h of transfection, cells were harvested and processed for immunoprecipitation and immunoblot as explained below.

### CELF1 immunoprecipitation, sample preparation and mass spectrometric analysis

The pEGFP plasmid encoding GFP-CELF1 (16) was transiently transfected into MCF-10A cells with TGF-β treatment using Lipofectamine LTX (ThermoFisher, Cat. 15338100) as per the manufacturer’s instructions. GFP-CELF1 was immunoprecipitated from MG-132 treated cells using GFP-trap nanobodies from Chromotek (Cat. gta). Immunoprecipitates from untreated MCF-10A cells with no MG-132 treatment were used as controls. Immunoprecipitates were resolved *via* SDS-PAGE, visualized by Coomassie Brilliant Blue, and the target protein region (molecular weight range 50kD to 120kD) was excised and eluted for enzyme digestion using Trypsin (GenDepot T9600) and Chymotrypsin (Promega V1061), separately. Ultra-high performance liquid chromatography-based mass spectrometry (MS) analysis was performed by Mass Spectrometry Proteomics core at Baylor College of Medicine, Houston TX. The post-digestion peptides were subjected to nanoflow LC-MS/MS analysis with a nano-LC 1200 system (Thermo Scientific) coupled to Orbitrap Fusion Lumos ETD (Thermo Scientific) mass spectrometer. The full MS was acquired in Orbitrap (120,000 resolution, 300–1400 m/z, 50 ms injection time, 5E5 AGC) and MS/MS in the IonTrap (Rapid scan, 1E4 AGC, 50 ms injection time) with CID fragmentation (30%). The dynamic exclusion was set to 15 s. The MS/MS spectra were searched against target-decoy human NCBI Refseq database (updated 2015_0610) in Proteome Discoverer 1.4 interface (Thermo Fisher) with Mascot algorithm (Mascot 2.4, Matrix Science). The precursor mass tolerance was confined within 20 ppm with fragment mass tolerance of 0.5 Da and a maximum of two missed cleavage allowed. Dynamic modification of oxidation on methionine, protein N-terminal acetylation, carbamidomethyl on cysteine, GlyGly on lysine, and phosphorylation on serine, threonine, and tyrosine was allowed. The peptides identified from the mascot result file were validated with a 5% false discover rate (FDR) and subject to manual verifications for correct assignment. Two biological replicates of the complete MS were performed and analyzed, with two sets of technical replicates in each repeat. Additional information is included in Fig. S1 and Table S1.

### Cell lysis, immunoprecipitations

Cells stably transduced with conditional expression vectors were treated with 0.1 μg/ml Doxycycline for 72 h and harvested at 80 to 90% confluency in Pierce IP Lysis/wash buffer (Cat. 87787). Protein lysates were quantified *via* BCA assay (Pierce: Cat. 23225). One mg of lysate was bound to 20 μl of washed Anti-FLAG M2 magnetic beads (Sigma, Cat. M8823) in IP lysis/wash buffer (500 μl total volume) overnight at 4 °C with rotation. The following day, beads were washed with lysis/wash buffer twice, boiled in 40 to 50 μl of 6× DTT Laemmli buffer, and analyzed *via* immunoblot.

For endogenous CELF1 and PKC-alpha/epsilon immunoprecipitation, MCF-10A cells −/+ TGF-β were harvested and protein lysates quantified as explained above. 5 μg of antibody was bound to 20 μl of Pierce Agarose A/G Plus beads (Cat. 20423) for 3 to 4 h with rotation at 4 °C. Afterward, 1 mg of protein lysate was bound to the bead-antibody complexes with rotation at 4 °C, overnight. The next day, beads were washed three times with lysis/wash buffer and processed for immunoblot.

### Immunoblots

All immunoblots were performed as previously described (16). Briefly, cell lysates were prepared in RIPA buffer from ThermoFisher Scientific (Cat. 89900) and quantified *via* BCA assay (Pierce: Cat. 23225). 30 to 40 μg of protein lysate was separated on NuPAGE 4 to 12% Bis-Tris protein gels (Cat. NP0322BOX) in MOPS SDS running buffer and transferred to the PVDF membrane (Immobilon-P, Millipore IPVH00010). The membrane was blocked in either 10% dry fat-free milk or 5% BSA, corresponding to the respective primary antibodies per manufacturer’s recommendation, in TBST. Following blocking, the membrane was incubated in primary antibody at 4 °C, overnight. Membranes were washed three times in 1× TBST for 10 min and incubated at room temperature for 1 h in secondary antibody. Membranes were again washed three times in TBST for 10 min each. For HRP-conjugated antibodies, the signal was determined *via* Pierce ECL Plus Western Blotting Substrate (Cat. 32132) and visualized on a 27,444 Bio-Rad ChemiDoc Touch. For Dylight secondary antibodies, the signal was visualized on a Li-COR Odyssey 9120. Antibodies used for immunoblots are catalogued in Table S2.

### Immunofluorescent staining

Cells were seeded on poly-D-lysine-coated 12 mm circle glass coverslips in 24-well plates. After fixation with 4% paraformaldehyde in PBS for 30 min, cells were permeabilized with 0.25% TritonX 100 in PBS for 10 min and blocked with 2% BSA in PBS for 30 min, all at room temperature and on a shaker. Cells were probed with indicated primary antibodies at 1:200 dilution in 2% BSA at 4 °C overnight. After three washes (PBS), secondary fluorescent antibodies: Alexa Fluor 488 (Jackson Immuno., Cat. AB_2338052) anti-rabbit, Alexa Fluor 647 anti-mouse (Jackson Immuno., Cat. AB_2338902) and Alexa Fluor 568 anti-mouse (Invitrogen, Cat. A-11004), were added at 1:1000 dilution in 2% BSA and incubated at room temperature for 1 h on the shaker. Cells were washed again three times with PBS, stained with Alexa Flour 647-labeled phalloidin (Cell Signaling Technologies Cat. #8940) for 15 min, washed once more in PBS, and mounted in DAPI-containing Vectashield (Vector Laboratories Cat. H-1200–10). Images were acquired on an ECHO Revolve FL or Nikon A1 confocal fluorescent microscope using the 40× objective lens and processed using ImageJ software.

### *In vitro* kinase assays

The ADP-glo Kinase assay kit from Promega (Catalog no. V6930) was used for *in vitro* kinase assays, following the manufacturer’s protocol. We used commercially purified (CusaBio) WT and PM CELF1 as substrate for kits for PKCα (Cat. V3381), PKCε (Cat. V4036) and GSK3β (Cat. V1991), respectively. A TECAN Infinity 200 was used for signal detection.

### Transwell migration and invasion assays

All assays were performed as described previously (Chaudhury *et al.*, 2016). Briefly, we used Cultrex R&D systems cell migration (Cat. 3465–096-K) and invasion (Cat. 3455–096-K) assay kits. Wells were coated with 0.1% BME 24 h prior to the experiment. Cells were treated with 10 μg/ml mitomycin C for 2 h, trypsinized, and 5 x 10^4^ cells in serum-free medium were added to each top chamber, with complete growth medium in the bottom wells of the plate. After 6 h (migration) or 24 h (invasion) migratory/invasive cells were stained with Calcein AM and fluorescence was detected on a TECAN Infinity 200 plate reader.

### 4T1 cell-derived xenograft experiments

Five-to six-week-old nude mice (NCRNU-EF-HOM, Taconic Biosciences) were used to study primary tumor development and metastasis for 4T1 cells stably transduced with inducible CELF1 knockdown/rescue vectors. Mice were acclimated for a week at the BCM mouse facility prior to injection of 1 × 10^5^ cells into the fourth mammary fat pad of each mouse. Mice were monitored post-surgery for primary tumor development. Beginning 5 days after injection, tumor size was measured every 2 days. When each experimental group reached the average tumor size of 200 mm^3^, the expression vectors were induced by adding 200 μg/ml doxycycline (DOX) into the mice’s drinking water. Fresh DOX water was given every 3 to 4 days. Primary tumors were surgically removed when reaching ∼1000 mm^3^. Metastatic burden was monitored every 2 to 4 days post-tumor removal *via* luciferase imaging on an IVIS lumina III with Living Image software (retro-orbital injections of 1 μg luciferin). Mice were humanely euthanized *via* CO_2_ inhalation upon a decline in animal health determined by breathing and motility. All animal experiments were performed under procedures and protocols approved by the Institutional Animal and Use Committee (IACUC) of Baylor College of Medicine.

### Statistical analyses

All data are presented with standard deviation (±). We used a two-tailed Student’s *t* test, two-way ANOVA analysis with a Tukey’s post-test, and the Mantel-Cox Log-rank test to calculate statistical significance, as appropriate. A *p*-value of less than 0.05 was considered statistically significant.

## Data availability

This manuscript contains mass spectrometric proteomics data that has been submitted to the ProteomeXchange Consortium *via* the PRIDE partner repository under the accession PXD035067.

## Supporting information

This article contains supporting information (25).

## Conflict of interest

The authors declare that they have no conflicts of interest with the contents of this article.
