# Supplementary material for: PKC-mediated phosphorylation governs the stability and function of CELF1 as a driver of EMT in breast epithelial cells
Source: J Biol Chem. 2024 Sep 27;300(11):107826. doi: 10.1016/j.jbc.2024.107826 (PMC11585768; doi:10.1016/j.jbc.2024.107826)
Supplement: Supplementary Figure 1 [file mmc1.pdf]

a

MAAFKLDFLPEMMVDHCSLNSSPVSKKMNGTLDHPDQPDLDAIKMFVGQVPRTWSEKDLRELFEQYGAVYEINVLR
DRSQNPPQSKGCCFVTFYTRKAALEAQNALHNMKVLPGMHHPIQMKPADSEKNNAVEDRKLFIGMISKKCTENDIR
VMFSSFGQIEECRILRGPDGLSRGCAFVTFTTRAMAQTAIKAMHQAQTMEGCSSPMVVKFADTQKDKEQKRMAQQ
LQQQMQQISAASVWGNLAGLNTLGPQYLALYLQLLQQTASSGNLNTLSSLHPMGGLNAMQLQNLAALAAAASAAQ
NTPSGTNALTT**SSSPLSVLTSSAGSSPSSSSSNS**VNPIASLGALQTLAGATAGLNVGSLAGMAALNGGLGSSGLSNGTGS
TMEALTQAYSGIQQYAAAALPTLYNQNLLTQQSIGAAGSQKEGPEGANLFIYHLPQEFGDQDLLQMFMPFGNVVSAK
VFIDKQTNLSKCFGFVSYDNPVSAQAAIQSMNGFQIGMKRLKVQLKRSKNDSKPY

b

T137- AMHQAQTMEGCSSPMVVK

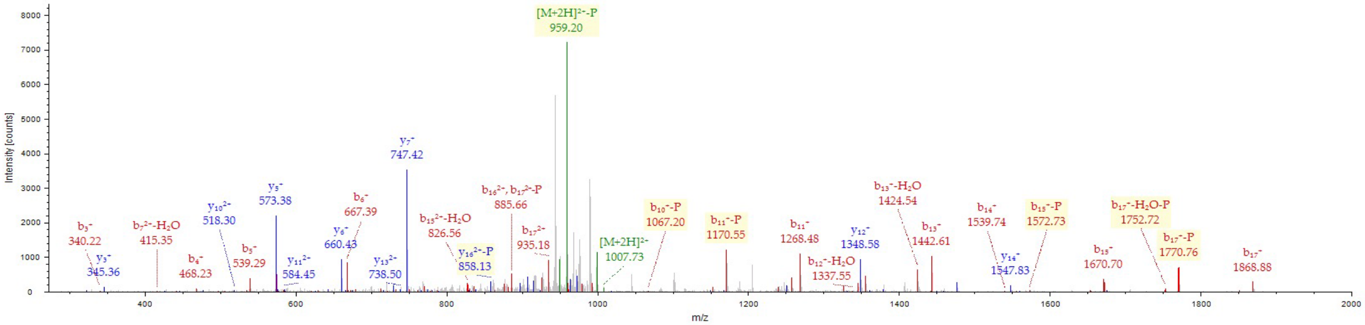

c

S285- AAASAAQNTPSGTNALTTSSSPLSVL

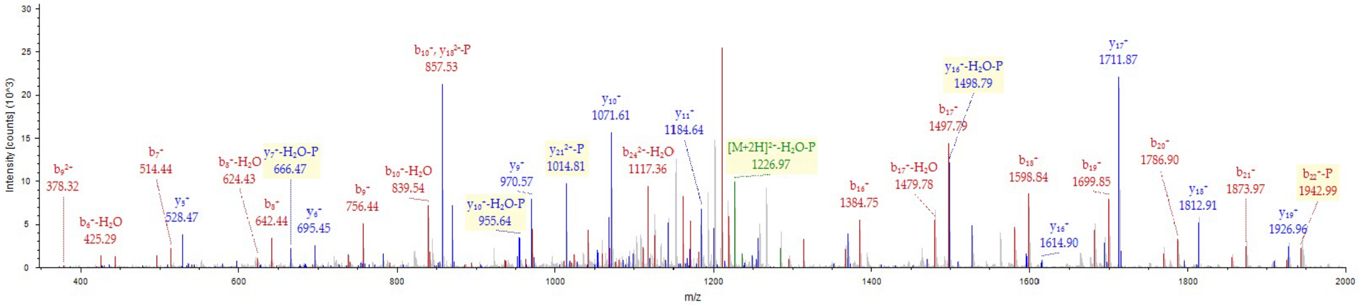

d

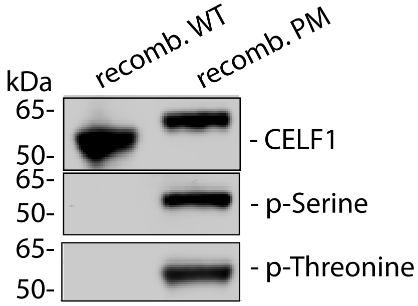

Supplementary figure 1: Mass spectrometric analysis of CELF1 and detection of phosphomimetic substitutions by immunoblot. **a.** CELF1 peptide sequence with lysine (K) and arginine (R) residues in red. Highlighted residues mark CELF1 region with lack of K and R residues for digestion, with SP-repeat dense region in bold. **b, c.** Representative spectra for two of the seven phosphorylation sites identified via mass-spectrometry, the identified site indicated in red in the coresponding peptide sequence above the graphs. **d.** Immunoblot of recombinant, bacterially expressed, purified CELF1 wild-type and phosphomimetic mutant proteins probed with anti-phosphoserine and anti-phosphothreonine antibodie as indicated. CELF1 serves as loading control. Data representative of a minimum of three replicates.
