# Supplementary material for: PKC-mediated phosphorylation governs the stability and function of CELF1 as a driver of EMT in breast epithelial cells
Source: J Biol Chem. 2024 Sep 27;300(11):107826. doi: 10.1016/j.jbc.2024.107826 (PMC11585768; doi:10.1016/j.jbc.2024.107826)
Supplement: Supplementary Figure 2 [file mmc2.pdf]

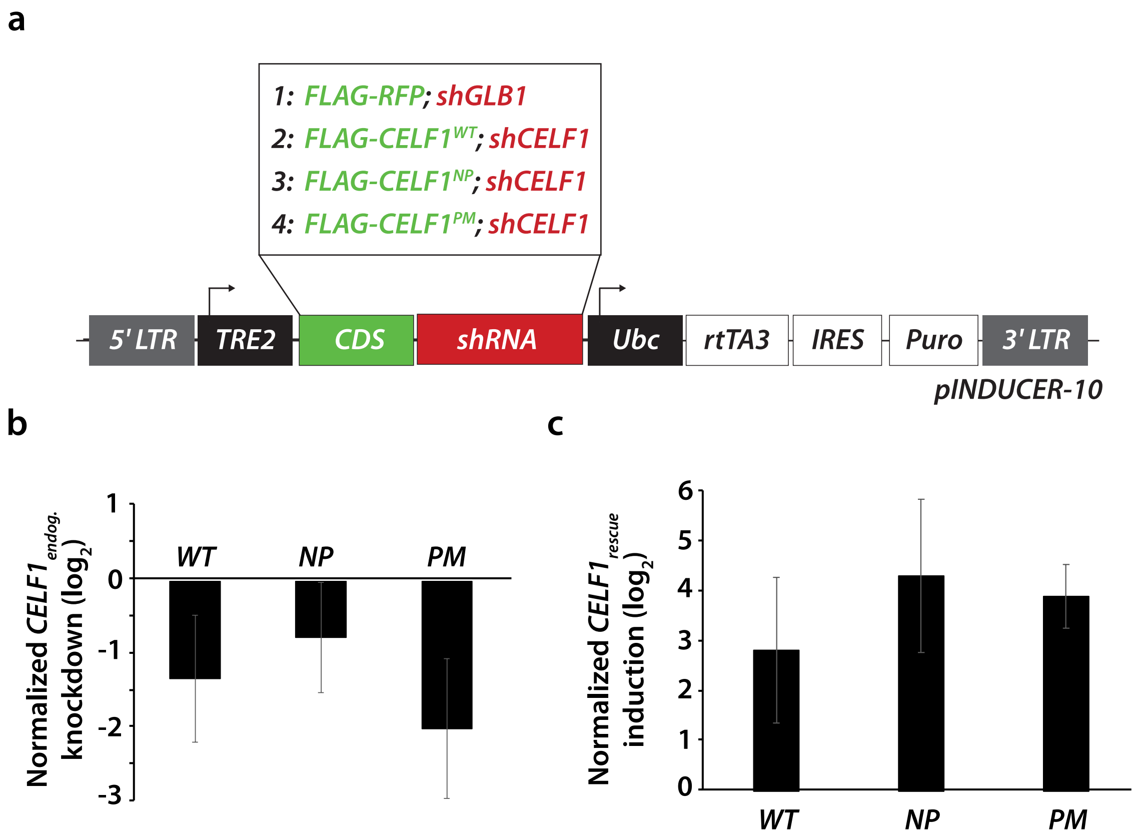

Supplementary Figure 2: Lentiviral Knockdown/Rescue System. a. The *pInducer-10* lentiviral expression vector (25) was modified for doxycycline-inducible knockdown of endogenous *CELF1* via the latter's 3' UTR concomitant with expression of *FLAG*-tagged wild-type *CELF1* (*WT*), *CELF1*-phosphomutant (*NP*), or *CELF1* phosphomimetic (*PM*) coding sequences. The control vector contains a *FLAG-RFP* coding sequence with an shRNA targeting beta-galactocidase (*shGLB1*). **b.** *TBP*-normalized ($^{-\Delta\Delta}C_t$) knockdown of endogenous *CELF1* mRNA in MCF-10A cells stably transduced with the indicated vector. Quantitative PCR was performed after 24 hours of treatment with 0.1 μg/mL doxycycline. Values are expressed relative to those derived from cells transduced with the *FLAG-RFP;shGLB1* vector. **c.** *TBP*-normalized ($^{-\Delta\Delta}C_t$) induction of vector-encoded *CELF1* mRNA in MCF-10A cells stably transduced with the indicated vector. Again, quantitative PCR was performed after 24 hours of treatment with 0.1 μg/mL doxycycline. Values are expressed relative to untreated cells, and vector-encoded *CELF1* was differentiated from endogenous *CELF1* via an amplicon including the the sequence encoding the fused FLAG affinity tag. Results for (**b**) and (**c**) representative of a minimum of three experiments performed in triplicate; error bars represent standard deviation.
