# Supplementary material for: PKC-mediated phosphorylation governs the stability and function of CELF1 as a driver of EMT in breast epithelial cells
Source: J Biol Chem. 2024 Sep 27;300(11):107826. doi: 10.1016/j.jbc.2024.107826 (PMC11585768; doi:10.1016/j.jbc.2024.107826)
Supplement: Supplementary Figure 3 [file mmc3.pdf]

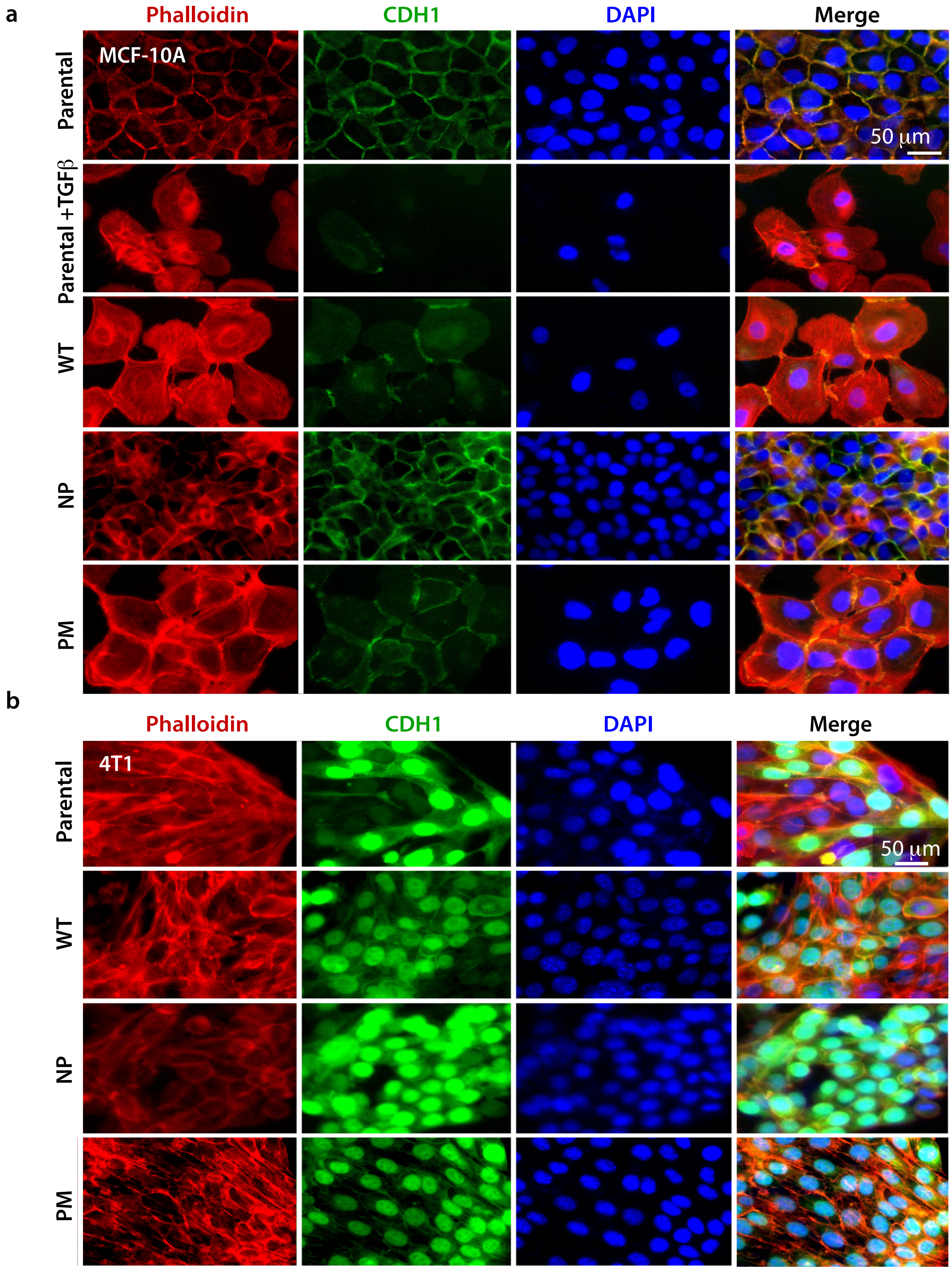

Supplementary Figure 3: Single channel representative images from Figure 3. Immunofluorescence of E-cadherin (CDH1, green), and actin (Phalloidin, red) subcellular distribution following induction of indicated knockdown-rescue constructs via treatment with 0.1 μg/ml doxycycline for 72 hours in: **a.** MCF-10A cells, where treatment with 5 ng/ml TGF-β treatment (also 72 hours) is used as a positive control, and **b**. 4T1 cells. DAPI nuclear counterstain is blue. Data representative of a minimum of three replicates.
