# Supplementary material for: PKC-mediated phosphorylation governs the stability and function of CELF1 as a driver of EMT in breast epithelial cells
Source: J Biol Chem. 2024 Sep 27;300(11):107826. doi: 10.1016/j.jbc.2024.107826 (PMC11585768; doi:10.1016/j.jbc.2024.107826)
Supplement: Supplementary Figure 4 [file mmc4.pdf]

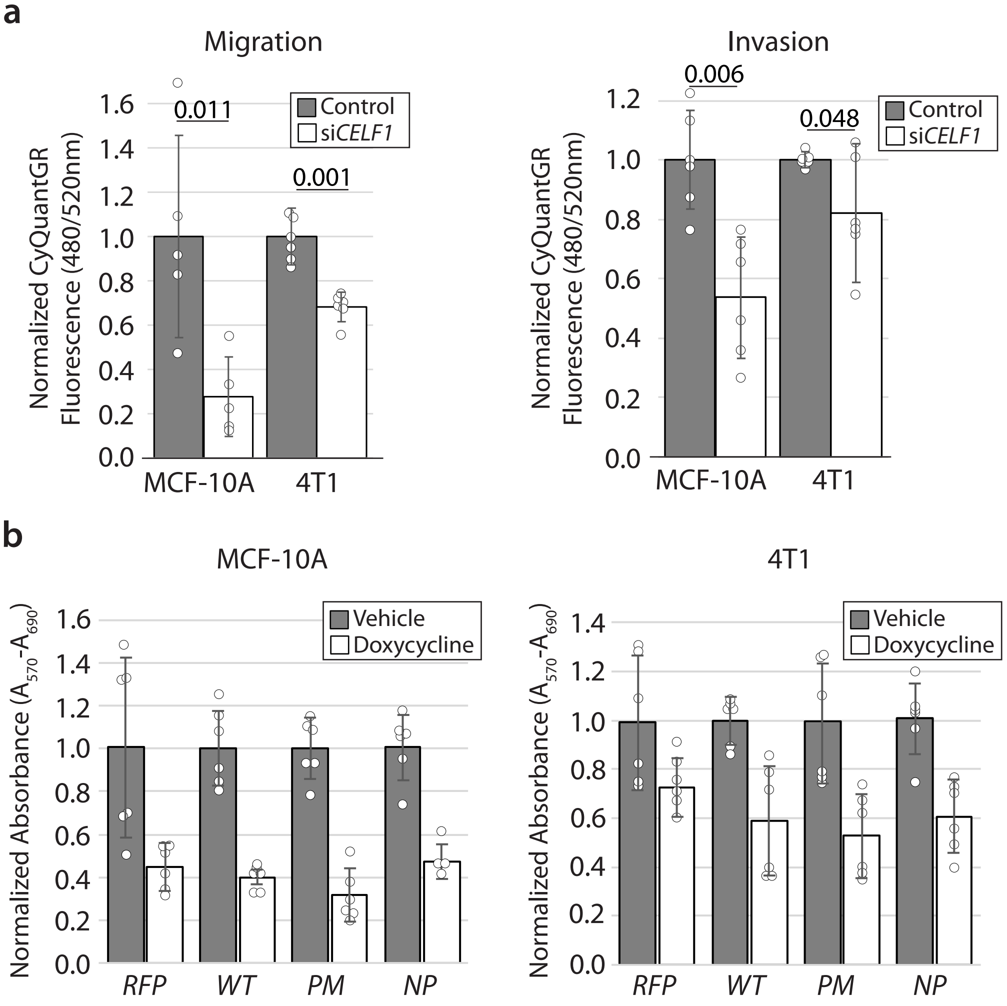

Supplementary Figure 4: CELF1 knockdown without rescue impedes migration and invasion, and knockdown/rescue sublines exhibit comparable viability and proliferation in migration and invasion assays. a. Migration (left) and invasion (right) of the indicated cell lines through transwells on a gradient of serum-free to complete growth medium for six or twelve hours, respectively, utilizing Sigma-Millipore's QCM Chemotaxis cell migration (Cat. #ECM509) and ECMatrix cell invasion (Cat. #ECM525) assays. Prior to the assay, MCF-10A sublines were plated at 5% confluency, treated with 5 ng/ml TGF-β and 0.1 μg/ml doxycycline for 72 hours, then serum starved (maintaining the presence of TGF-β and doxycycline) for 24 hours, trypsinized, counted, and plated and analyzed as per the manufacturer's recommendations. 4T1 sublines were processed identically, minus the inclusion of TGF-β. Control denotes the *FLAG-RFP;shGLB1* subline for MCF-10A cells and the parental 4T1 line; *siCELF1* denotes sublines stably transduced with *pInducer-10* vectors encoding a doxycycline-inducible *FLAG-RFP;shCELF1* expression construct. **b.** Production of MTT formazan by MCF-10A (left) and 4T1 (right) sublines treated as in (**a**) as a proxy for migration and invasion assays. Briefly, sublines were again plated at 5% confluency, and treated with 5 ng/ml TGF-β (MCF-10A only) with or without 0.1 μg/mL doxycycline induction for 72 hours, then serum starved (maintaining the presence or absence of TGF-β and doxycycline) for 24 hours and switched to growth medium with 6.4% serum (to mimic the the aggregate serum concentration in the transwell assays) for 24 hours . MTT reagent (Sigma-Aldrich Cat. #CGD1) was then added to the cultures for two hours, and MTT formazan crystals were solublized and read on a plate reader as per the manufacturer's instructions. Normalized relative results were essentially equivalent for sublines retaining complete growth medium with or without doxycycline for the duration of analagous experiments (data not shown). Doxycycline vehicle was 50:50 ddH_2O:DMSO. All results in (**a**) and (**b**) depict the aggregate of a minimum of two independent experiments performed in triplicate, error bars represent standard deviation for the aggregate. P values indicated, Student's paired two-tailed t test.
