# Supplementary material for: PKC-mediated phosphorylation governs the stability and function of CELF1 as a driver of EMT in breast epithelial cells
Source: J Biol Chem. 2024 Sep 27;300(11):107826. doi: 10.1016/j.jbc.2024.107826 (PMC11585768; doi:10.1016/j.jbc.2024.107826)
Supplement: Supplementary Figure 5 [file mmc5.pdf]

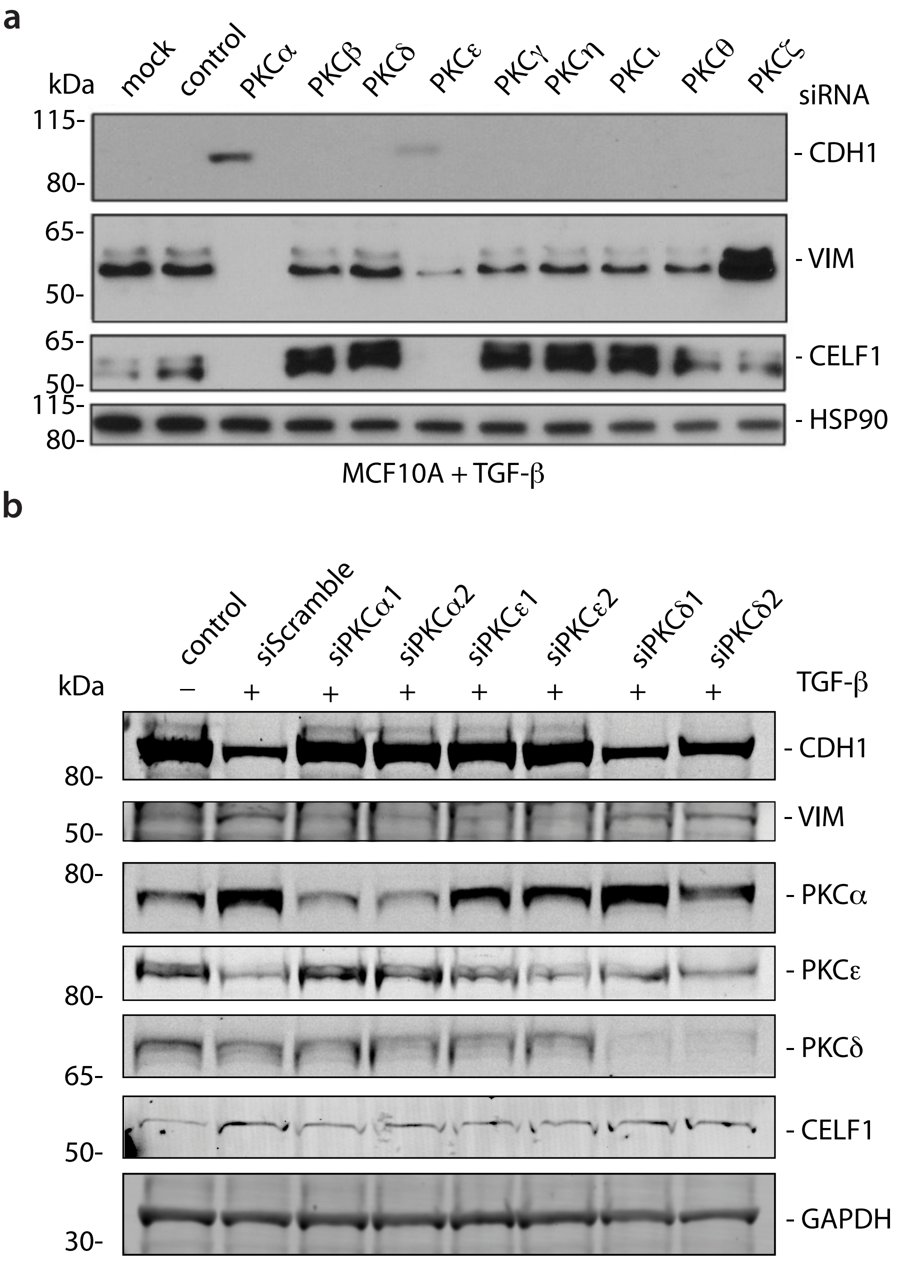

Supplementary Figure 5: Protein Kinase C family siRNA knockdown screen and secondary validation. **a.** Immunoblot analysis of relative E-cadherin, (CDH1), vimentin (VIM) and CELF1 protein expression in MCF10A cells following transfection of 20 nM of the indicated siRNAs and treatment with 5 ng/mL TGF-β for 72 hours. Mock is a mock transfection, control is a scrambled control siRNA. HSP90 is a loading control. **b.** Experimental repeat of Figure 4a separately utilizing the two distinct siRNAs in one experimental replicate. Again, cells were transfected with 20 nM of the indicated siRNAs and treated with 5 ng/mL TGF-β for 72 hours. GAPDH is a loading control. All data representative of a minimum of three experimental replicates with two distinct siRNAs.
