# Supplementary material for: PKC-mediated phosphorylation governs the stability and function of CELF1 as a driver of EMT in breast epithelial cells
Source: J Biol Chem. 2024 Sep 27;300(11):107826. doi: 10.1016/j.jbc.2024.107826 (PMC11585768; doi:10.1016/j.jbc.2024.107826)
Supplement: Supplementary Figure 6 [file mmc6.pdf]

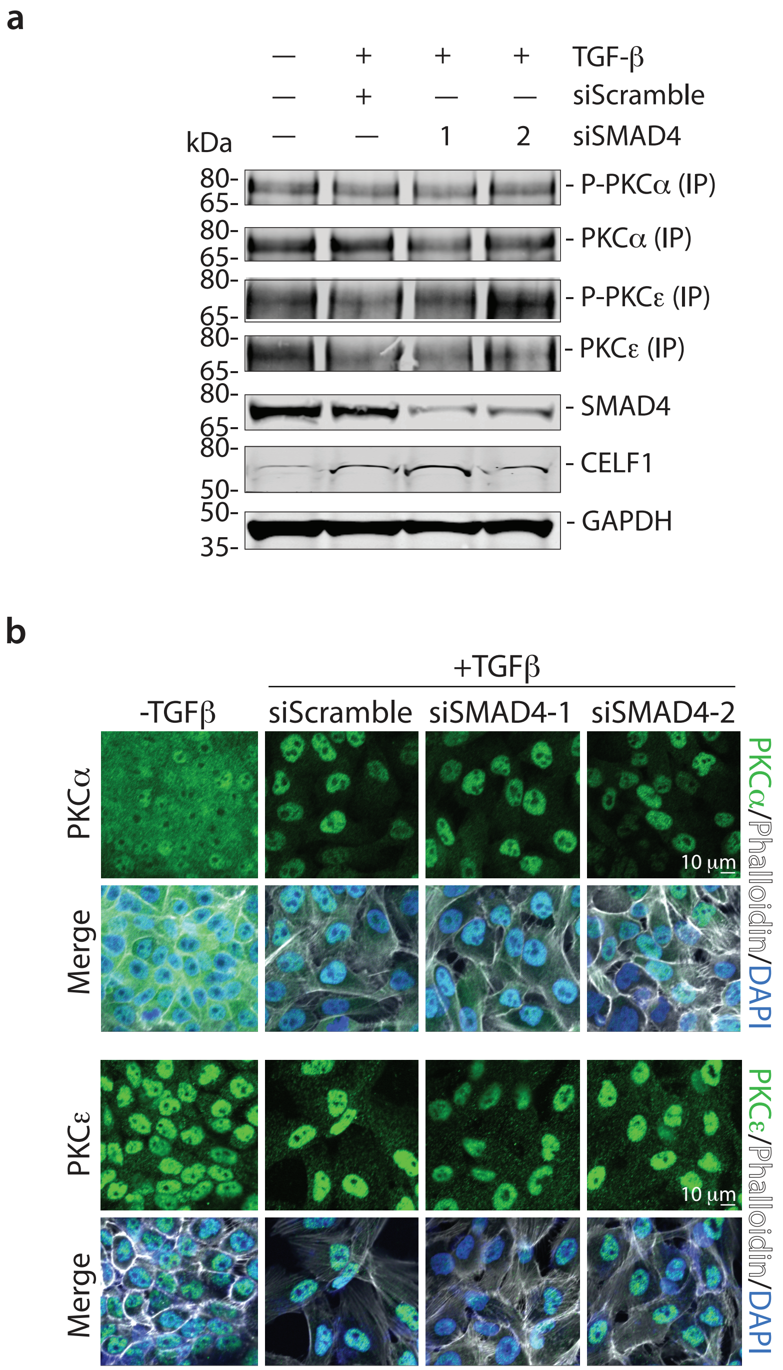

Supplementary Figure 6: PKC activation, CELF1 stabilization, and PKC subcellular localization are independent of SMAD4 activity. a. Experimental repeat of Figure 4e separately utilizing the two distinct siRNAs in one experimental replicate, demonstrating that SMAD4 knockdown disrupts neither PKC activation nor CELF1 stabilization. GAPDH is a loading control **b.** Experimental repeat of Figure 4f separately utilizing the two distinct siRNAs in one experimental replicate, demonstrating that PKCα localizes to the nucleus upon TGF-β treatment, and this is not impacted by SMAD4 knockdown. All data representative of a minimum of three experimental replicates with two distinct siRNAs.
