# Supplementary material for: PKC-mediated phosphorylation governs the stability and function of CELF1 as a driver of EMT in breast epithelial cells
Source: J Biol Chem. 2024 Sep 27;300(11):107826. doi: 10.1016/j.jbc.2024.107826 (PMC11585768; doi:10.1016/j.jbc.2024.107826)
Supplement: Supplementary Figure 7 [file mmc7.pdf]

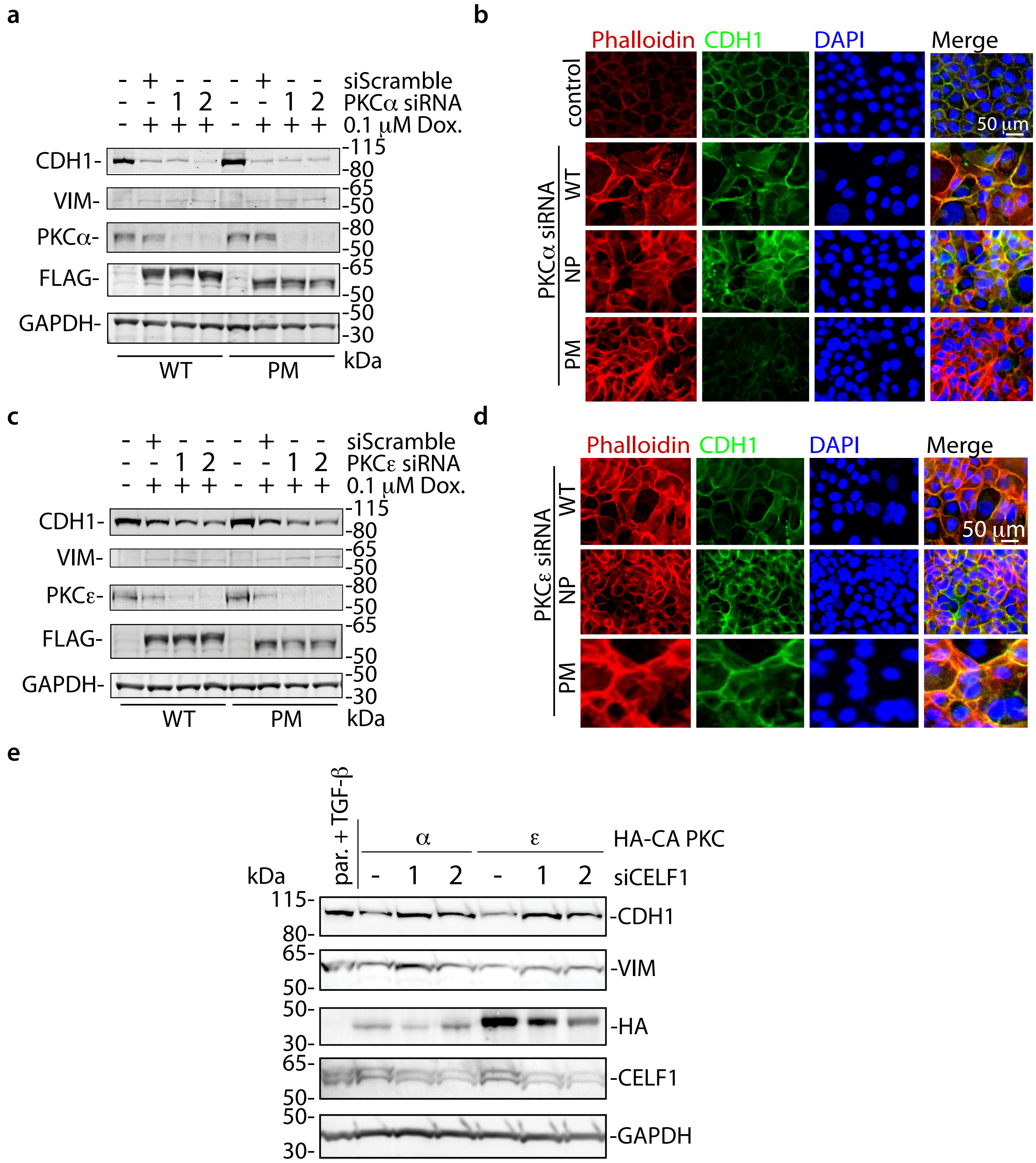

Supplementary Figure 7: a. Experimental repeat of Figure 5a separately utilizing the two distinct siRNAs in one experimental replicate. Cells were transfected with 20 nM of the indicated siRNA and then induced with doxycycline for 72 hours. GAPDH is a loading control. **b.** Single channel representative images from Figure 5b. Immunofluorescence of E-cadherin (CDH1, green), and actin (Phalloidin, red) subcellular distribution following siRNA-mediated knockdown (20 nM) of PKCα and induction of the indicated CELF1 variants via treatment of stably transduced MCF10A sublines with 0.1 μg/mL doxycycline for 72 hours. DAPI nuclear counterstain is blue. **c., d.** As (a) and (b) , knocking down PKCε. **e.** Experimental repeat of Figure 5e separately utilizing the two distinct siRNAs in one experimental replicate. Again, GAPDH is a loading control. All data representative of a minimum of three replicates with two distinct siRNAs.
