# Supplementary material for: PKC-mediated phosphorylation governs the stability and function of CELF1 as a driver of EMT in breast epithelial cells
Source: J Biol Chem. 2024 Sep 27;300(11):107826. doi: 10.1016/j.jbc.2024.107826 (PMC11585768; doi:10.1016/j.jbc.2024.107826)
Supplement: Supplementary Figure 8 [file mmc8.pdf]

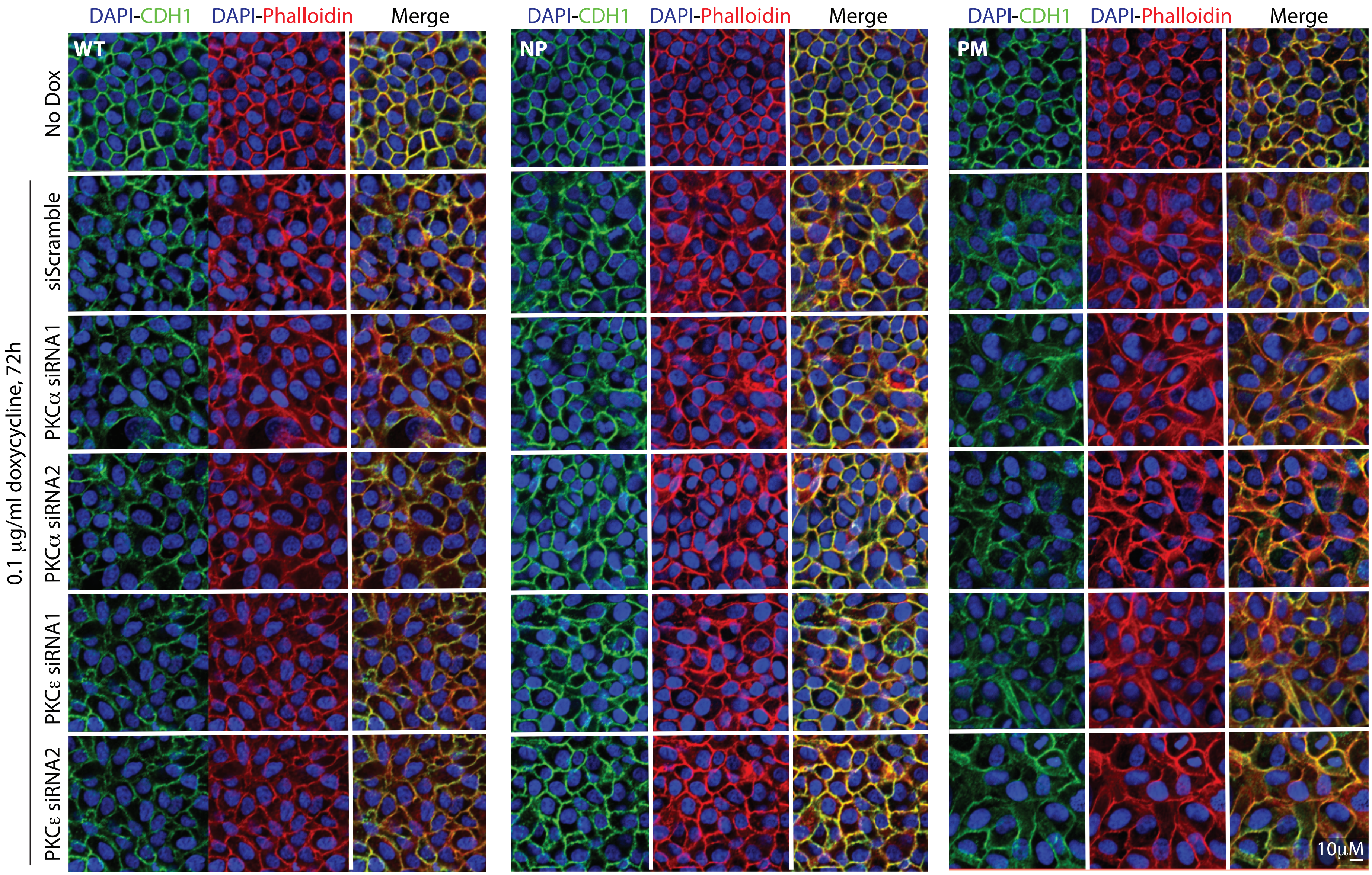

Supplementary Figure 8: Full representative dataset of E-cadherin (CDH, green) and actin (Phalloidin, red) subcelluar distribution in stably transduced and puromycin-selected MCF-10A cell lines using two distinct siRNAs. The cell lines were transfected wth 20 nM of the indicated siRNAs, and the following day the indicated CELF1 variants were induced with 0.1 μg/mL doxycycline for 72 hours. No Dox = No treatment. DAPI nuclear counterstain is blue. Images collected using the 40X oil immersion objective lens on a Nikon A1 confocal microscope and processed on ImageJ software. Data representative of a minimum of four individual experiments.
