# Supplementary material for: PKC-mediated phosphorylation governs the stability and function of CELF1 as a driver of EMT in breast epithelial cells
Source: J Biol Chem. 2024 Sep 27;300(11):107826. doi: 10.1016/j.jbc.2024.107826 (PMC11585768; doi:10.1016/j.jbc.2024.107826)
Supplement: Supplementary Figure 9 [file mmc9.pdf]

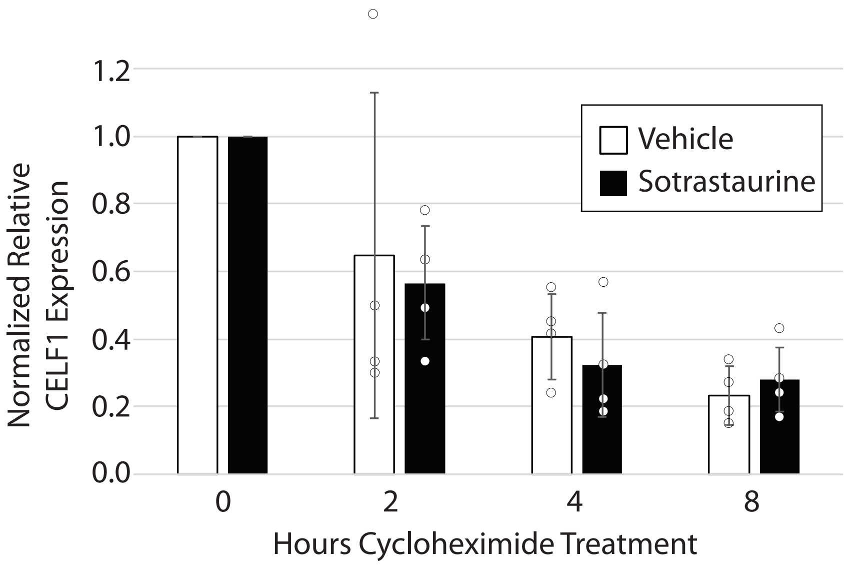

Supplementary Figure 9: Pharmacologic inhibition of PKC activity does not impact the stability of an existing pool of phosphorylated CELF1. MCF-10A cells were treated for 72 hours with 5 ng/ml TGF-β, then treated with 40 μg/ml cycloheximide in the presence or absence of 0.5 μM of the PKC inhibitor Sotrastaurine. Vehicle is DMSO. Cellular extracts were collected at the indicated time points and immunoblotted for both CELF1 and GAPDH expression. Blots were quantified via ImageJ, and relative CELF1 band density at each time point was normalized to relative GAPDH band density, setting this ratio at the beginning of cycloheximide treatment to 1. Graph depicts the aggregate data derived from four experimental replicates, error bars depict standard deviation.
