# Supplementary material for: PKC-mediated phosphorylation governs the stability and function of CELF1 as a driver of EMT in breast epithelial cells
Source: J Biol Chem. 2024 Sep 27;300(11):107826. doi: 10.1016/j.jbc.2024.107826 (PMC11585768; doi:10.1016/j.jbc.2024.107826)
Supplement: Supplementary Table 1 [file mmc10.pdf]

Supplementary Table 1: Abbreviated Mass Spectrometry Data for Identified Phosphorylation Sites

Peptide sequence	PTM site	Peptide Modified Sequence
AMHQAQTMEGCSSPMVVK	T173	AMHQAQT[+80]MEGCSSPMVVK
AMHQAQTMEGCSSPMVVK	S178	AMHQAQTMEGCS[+80]SPMVVK
AAAASAAQNTPSGTNALTTSSSPLSVL	S285/286	AAAASAAQNTPSGTNALTTSSS[+80]PLSVL
SVLTSSGSPSSSSSNSVNPIASL	S295/296/298	SVLTSSGSS[+80]PSSSSSNSVNPIASL

Precursor Charge	mass/charge	Ion score	Ions matched
2	1007.90997	52	11/192
2	1044.42981	40	16/192
2	1284.60864	49	23/260
2	1166.5271	58	14/206
