# Supplementary material for: PKC-mediated phosphorylation governs the stability and function of CELF1 as a driver of EMT in breast epithelial cells
Source: J Biol Chem. 2024 Sep 27;300(11):107826. doi: 10.1016/j.jbc.2024.107826 (PMC11585768; doi:10.1016/j.jbc.2024.107826)
Supplement: Supplementary Table 2 [file mmc11.pdf]

Supplementary Table 2: List of antibodies used for immunoblots and immunofluorescence.

Antibody name	Species	Vendor	Catalog no.	Dilution
CELF1	mouse	Abcam	ab9549	1:1,000
E-cadherin	rabbit	Cell signaling Technology	3195	1:2,000
FLAG-tag (M2)	mouse	Sigma	F1804	1:2,000
Vimentin	mouse	Abcam	ab8978	1:1,000
SMAD4	rabbit	Cell signaling Technology	38454	1:1,000
HA-tag	rabbit	Cell signaling Technology	3724	1:1,000
GAPDH	mouse	EMD Millipore	MAB374	1:2,000
HSP90	mouse	BD transduction	610419	1:1,000
phosphoserine	rabbit	Abcam	ab9332	1:1,000
phosphothreonine	rabbit	Abcam	ab9337	1:1,000
phosphotyrosine	mouse	Cell signaling Technology	9411S	1:1,000
PKC alpha	rabbit	Cell signaling Technology	2056S	1:1,000
PKC delta	rabbit	Cell signaling Technology	2055	1:1,000
PKC epsilon	rabbit	Cell signaling Technology	9616	1:1,000
Phospho-PKC (pan)	rabbit	Cell signaling Technology	38938	1:1,000
Peroxidase IgG anti-mouse	goat	Jackson ImmunoResearch laboratories	115-035-174	1:10,000
Peroxidase IgG anti-rabbit	rabbit	Jackson ImmunoResearch laboratories	211-032-171	1:10,000
DyLight-680 anti-mouse	goat	Cell signaling Technology	5470S	1:12,000
DyLight-800 anti-rabbit	mouse	Cell signaling Technology	5151S	1:12,000
AF555 anti-rabbit	donkey	Jackson ImmunoResearch laboratories	711-565-152	1:1000
AF488 anti-mouse	goat	Jackson ImmunoResearch laboratories	115-545-071	1:1000
AF647 phalloidin	N/A	Cell signaling Technology	8940	330 nM
